# A complex of C9ORF72 and p62 uses arginine methylation to eliminate stress granules by autophagy

**DOI:** 10.1038/s41467-018-05273-7

**Published:** 2018-07-18

**Authors:** Maneka Chitiprolu, Chantal Jagow, Veronique Tremblay, Emma Bondy-Chorney, Geneviève Paris, Alexandre Savard, Gareth Palidwor, Francesca A. Barry, Lorne Zinman, Julia Keith, Ekaterina Rogaeva, Janice Robertson, Mathieu Lavallée-Adam, John Woulfe, Jean-François Couture, Jocelyn Côté, Derrick Gibbings

**Affiliations:** 1 Department of Cellular and Molecular Medicine, University of Ottawa, 451 Smyth Road, Ottawa, Ontario K1H 8M5 Canada; 2 Ottawa Institute of Systems Biology, University of Ottawa, 451 Smyth Road, Ottawa, Ontario K1H 8M5 Canada; 3 Department of Biochemistry, Microbiology and Immunology, University of Ottawa, 451 Smyth Road, Ottawa, Ontario K1H 8M5 Canada; 4 Ottawa Bioinformatics Core Facility, Ottawa Hospital Research Institute, 501 Smyth Road, Ottawa, Ontario K1H 8L6 Canada; 5 Sunnybrook Health Sciences Centre, 2075 Bayview Avenue, Toronto, Ontario M4N 3M5 Canada; 6 Tanz Centre for Research in Neurodegenerative Disease, University of Toronto, Krembil Discovery Tower, 60 Leonard Avenue, Toronto, Ontario M5T 2S8 Canada; 7 Department of Pathology and Laboratory Medicine, University of Ottawa, 501 Smyth Road, Ottawa, Ontario K1H 8L6 Canada

## Abstract

Mutations in proteins like FUS which cause Amyotrophic Lateral Sclerosis (ALS) result in the aberrant formation of stress granules while ALS-linked mutations in other proteins impede elimination of stress granules. Repeat expansions in C9ORF72, the major cause of ALS, reduce C9ORF72 levels but how this impacts stress granules is uncertain. Here, we demonstrate that C9ORF72 associates with the autophagy receptor p62 and controls elimination of stress granules by autophagy. This requires p62 to associate via the Tudor protein SMN with proteins, including FUS, that are symmetrically methylated on arginines. Mice lacking p62 accumulate arginine-methylated proteins and alterations in FUS-dependent splicing. Patients with C9ORF72 repeat expansions accumulate symmetric arginine dimethylated proteins which co-localize with p62. This suggests that C9ORF72 initiates a cascade of ALS-linked proteins (C9ORF72, p62, SMN, FUS) to recognize stress granules for degradation by autophagy and hallmarks of a defect in this process are observable in ALS patients.

## Abstract (web summary)

Many Amyotrophic Lateral Sclerosis (ALS)-linked mutations cause accumulation of stress granules, and most ALS cases are caused by repeat expansions in C9ORF72. Here the authors show that C9ORF72 and the autophagy receptor p62 interact to associate with proteins symmetrically dimethylated on arginines such as FUS, to eliminate stress granules by autophagy.

## Introduction

In autophagy, a phagophore elongates to engulf and then enclose cytoplasmic contents in an autophagosome. Fusion of the autophagosome with lysosomes generates an autophagolysosome and results in cargo degradation^1^. Cargoes are frequently recruited for degradation by autophagy in a selective manner. Autophagy receptors such as p62 (Sequestosome-1 (SQSTM1)) bind both specific cargoes and LC3, an integral part of the elongating phagophore, thereby directing the selective recruitment of cytoplasmic cargoes into autophagosomes^2,3^. In most published cases ubiquitination of substrates is required for their recognition by selective autophagy receptors, but in some cases degradation appears to be independent of ubiquitination^4^.

Early studies demonstrated that a large proportion of RNA degradation in stress is performed by autophagy^5^. It is now increasingly clear that cytoplasmic RNA granules, including stress granules, are degraded by autophagy^6–8^. Stress granules coalesce RNA and RNA-binding proteins in large cytoplasmic clusters within minutes of stresses such as oxidative stress^9,10^. When stressors are removed, many stress granules disassemble, but a significant proportion relies on autophagy for their elimination^7^.

The rapid concentration of select RNA-binding proteins controlling splicing and translation in stress granules is postulated to re-shape the post-transcriptional landscape to rapidly tailor cellular responses to stress^9,10^. Interestingly, several mutations genetically linked to ALS impair nuclear localization of RNA-binding proteins like FUS and TDP-43 causing them to accumulate in cytoplasmic stress granules and insoluble inclusions in patient neurons^11^. Importantly, evidence suggests that pathology in ALS is caused not only by loss of the splicing capacity in the nucleus of proteins like FUS, but also by a toxic gain of function in the cytoplasm^12^. This has led to a model in which inappropriate induction of stress granules or their derivatives may play a role in ALS pathology^13^.

Intriguingly, mutations in valosin-containing protein (VCP), which are an inherited cause of ALS, control elimination of stress granules by autophagy^7^. P62 localizes to many types of inclusions in ALS patients. We previously demonstrated that p62, which is genetically linked to ALS^14^, degrades stress granule-like cytoplasmic aggregates by selective autophagy^6^. This suggests that either instigating formation of inclusions related to stress granules or impeding their clearance by selective autophagy may have a role in ALS.

Repeat expansions in an intron of C9ORF72 are the most common genetic cause of ALS^15,16^. Repetitive RNAs and dipeptide repeat proteins are produced by transcription and translation of these repeat expansions and overexpression of these can induce ALS-like pathology^17^. C9ORF72 repeat expansions also cause decreased levels of C9ORF72 mRNA and protein, suggesting that alongside repeat-induced pathology certain aspects of ALS pathology could be caused by loss of C9ORF72 function^18,19^.

Here, we show that symmetric arginine methylation of stress granules by Protein Arginine Methyltransferase 5 (PRMT5) is required for a complex of C9ORF72 and p62 to associate with stress granules and eliminate them by autophagy. Additionally, *p62*^−/−^ mice and patients with C9ORF72 repeat expansions show signs of defects in this process.

## Results

### C9ORF72 associates with stress granule proteins and p62

**Fig. 1:**
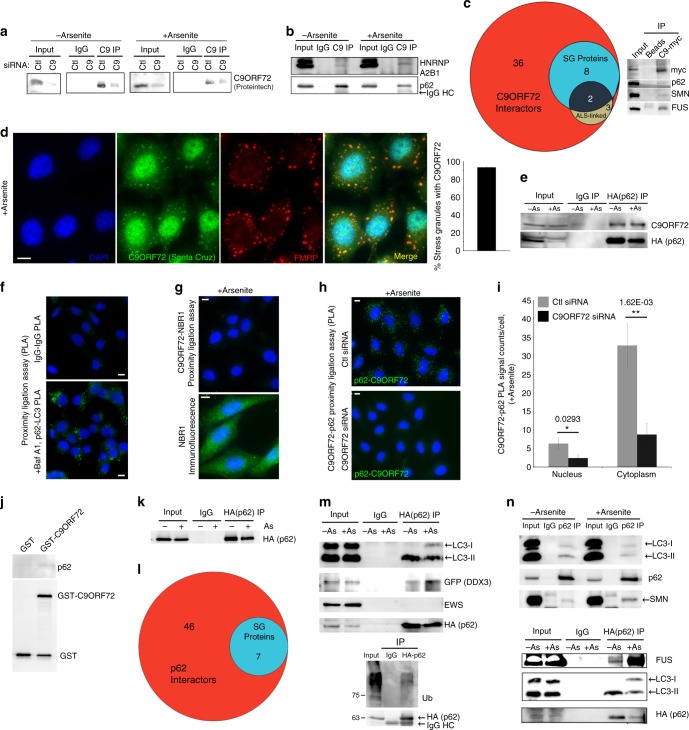
C9ORF72 associates with ALS-linked stress granule proteins including p62. **a** Western blot of endogenous C9ORF72 immunoprecipitates compared to immunoprecipitation with non-specific IgG from cell lysates (input) transfected with control or C9ORF72 siRNA. Secondary antibody specific for IgG light chain was used. **b** Western blot of endogenous C9ORF72 immunoprecipitates with and without arsenite treatment. **c** Left, Venn-diagram depicting the number of proteins in the putative C9ORF72 interactome that localize to stress granules (SGs, blue) or contain mutations linked to ALS (olive); Right, western blot of immunoprecipitation of C9ORF72-myc for p62 and SG proteins (FUS, SMN). **d** Immunofluorescence of endogenous C9ORF72 and SGs (FMRP) after 30 min incubation with Sodium Arsenite, (0.5 mM); Right, quantification of the percentage of SGs (FMRP+) co-localizing with foci of C9ORF72 (quantified 3916 C9ORF72 punctae and 3331 SGs). **e** Western blot of HA-p62 immunoprecipitates with and without arsenite treatment. **f** PLA co-labeling with species controlled IgG (top, negative control) and known interactors p62 and LC3 (bottom, positive control); p62-LC3 association was tested in cells treated with Bafilomycin A1 (Baf A1). **g** Top, proximity ligation assay (PLA) for C9ORF72 and NBR1 in arsenite-treated cells; Bottom, immunofluorescence for NBR1 showing antibody does label cells. **h** PLA for endogenous p62 and C9ORF72 in cells treated with arsenite and transfected with control (Ctl) or C9ORF72 (C9) siRNA. **i** Quantification of PLA signals for C9ORF72 and p62 in the cytoplasm and nucleus in identical conditions to (**h**) (*n* = 3, mean ± SD, Student’s *t*-test). **j** Western blot of recombinant p62 and GST-C9ORF72 from in vitro pulldown assay. **k** Representative immunoprecipitation of HA-p62 used for identification of putative p62 interacting proteins using LC-MS/MS. **l** Venn-diagram depicting number of proteins in predicted p62 interactome that localize to SGs (blue). **m** Western blot of immunoprecipitates of HA-p62 detecting known interactors (LC3, ubiquitinated proteins), candidate interactors identified by LC-MS/MS (GFP-DDX3) and control RNA-binding proteins that do not associate with p62 (EWS) in cells treated or not with arsenite. **n** Western blot of immunoprecipitations of endogenous p62 (top) and HA-p62 (bottom) for other SG proteins (FUS, SMN). HeLa cells were used in all experiments. Scale bar = 10 µm. Molecular weight markers for blots, where not shown, are included in the uncropped versions

To identify new functions of C9ORF72 during oxidative stress, we immunoprecipitated endogenous C9ORF72 from cells exposed to arsenite-induced oxidative stress (+As) and untreated counterparts (−As, Fig. 1a). C9ORF72 was detected as expected at 50 kDa with a secondary antibody specific for IgG light chain (Fig. 1a). This band was diminished in cells treated with a C9ORF72 siRNA (Fig. 1a, Supplementary Fig. 1a, b) confirming the specificity of the immuno-enrichment. C9ORF72 immunoprecipitated a known C9ORF72 interactor (hnRNPA2B1) (Fig. 1b). Mass spectrometry of three biological replicates of C9ORF72 immunoprecipitates compared to IgG controls identified 309 proteins putatively interacting with C9ORF72 (Bayesian False Discovery Rate < 0.05) as assessed by the Significance Analysis of INTeractome (SAINT) algorithm (Supplementary Fig. 1c, Supplementary Table 1). Stringently limiting candidates to those enriched in C9ORF72 immunoprecipitation vs. IgG control in each of the three biological replicates yielded 36 high-confidence interactors including multiple previously identified C9ORF72 interactors (Fig. 1c, Supplementary Table 1). Strikingly, 8 (22%) of the C9ORF72 interactors during oxidative stress were known stress granule components^20–22^ including two ALS-linked proteins (hnRNPA2B1 and hnRNPA1, Fig. 1c, Supplementary Table 1). FUS and SMN, two other ALS-linked proteins that localize to stress granules, also immunoprecipitated with C9ORF72-myc (Fig. 1c, Supplementary Fig. 1d, e). Putative C9ORF72 interactors were enriched in RNA binding and RNA splicing GO terms (Supplementary Table 2). Upon exposure to oxidative stress induced by arsenite, multiple RNA-binding proteins including PABP, TIAR, FMRP, FUS, SMN and DDX3 were recruited into stress granules (Supplementary Fig. 1d, e). Consistent with putative interactions of C9ORF72 with stress granules, C9ORF72 was recruited into 93% of FMRP + stress granules (Fig. 1d, Supplementary Fig. 1f, g). This suggests that C9ORF72 has a role in stress granule biology.

Intriguingly, a third ALS-linked protein that is a high-confidence candidate interactor with C9ORF72 is p62 (Supplementary Table 1). C9ORF72 immunoprecipitated p62, and HA-p62 reciprocally immunoprecipitated C9ORF72 both in the absence and presence of oxidative stress (Fig. 1a, b, e). Proximity ligation assays (PLA) confirmed that in intact cells p62 and C9ORF72 closely associate. While no PLA signal was detected with control non-specific antibodies (Fig. 1f), or between C9ORF72 and another autophagy receptor NBR1 (Fig. 1g), prominent PLA signals were obtained between p62 and a known interactor (LC3) as well as between C9ORF72 and p62 (Fig. 1f, h). PLA signal between C9ORF72 and p62 was lost upon knockdown of C9ORF72 (Fig. 1h, i). Finally, more recombinant p62 was pulled down with recombinant purified GST-C9ORF72 than with beads containing GST alone (Fig. 1j). Together, this suggests that C9ORF72 and p62 are part of a physically-associated complex.

We investigated whether C9ORF72 and p62 have a shared role in stress granule biology. We immunoprecipitated HA-p62 (Fig. 1k). p62 interactors (vs. IgG control immunoprecipitates) with and without oxidative stress were identified by mass spectrometry analysis using SAINT (Supplementary Table 3, Fig. 1l). In addition to immunoprecipitating LC3 and ubiquitinated proteins as anticipated, p62 immunoprecipitated candidate interactor DDX3, but not EWS another RNA-binding protein (Fig. 1m). Candidate p62 interactors overlapped with putative p62 interactors from other studies curated in iRefWeb^23^ by 26.3% (20/76 proteins) in untreated cells and 26.1% (12/46 proteins) in arsenite-treated cells compared to random overlap of 2.9% (431 putative interactors/15,000 proteins in the proteome). Like C9ORF72, candidate p62 interactors were strongly enriched in RNA splicing functions and 29% of p62 interacting proteins were RNA-binding proteins (Supplementary Table 4). Seven (15%) of the candidate p62 interactors were known stress granule components (Fig. 1l, Supplementary Table 5). p62 also interacted with SMN and other RNA-binding proteins including FUS (Fig. 1n), which localize to stress granules (Supplementary Fig. 1d, e). p62 interaction with FUS was independent of RNA (Supplementary Fig. 1h, i). Strikingly, p62 and C9ORF72 interactomes upon oxidative stress significantly overlapped (*p* < 0.0001, Chi-test) including RNA-binding proteins known to localize to stress granules (Supplementary Table 6). This strongly suggests that a complex of C9ORF72 and the autophagy receptor p62 (Fig. 1b, e–j, Supplementary Table 1) associates with stress granule proteins in cells exposed to oxidative stress (Fig. 1b–d, k–n).

### C9ORF72 and p62 help eliminate stress granules by autophagy

**Fig. 2:**
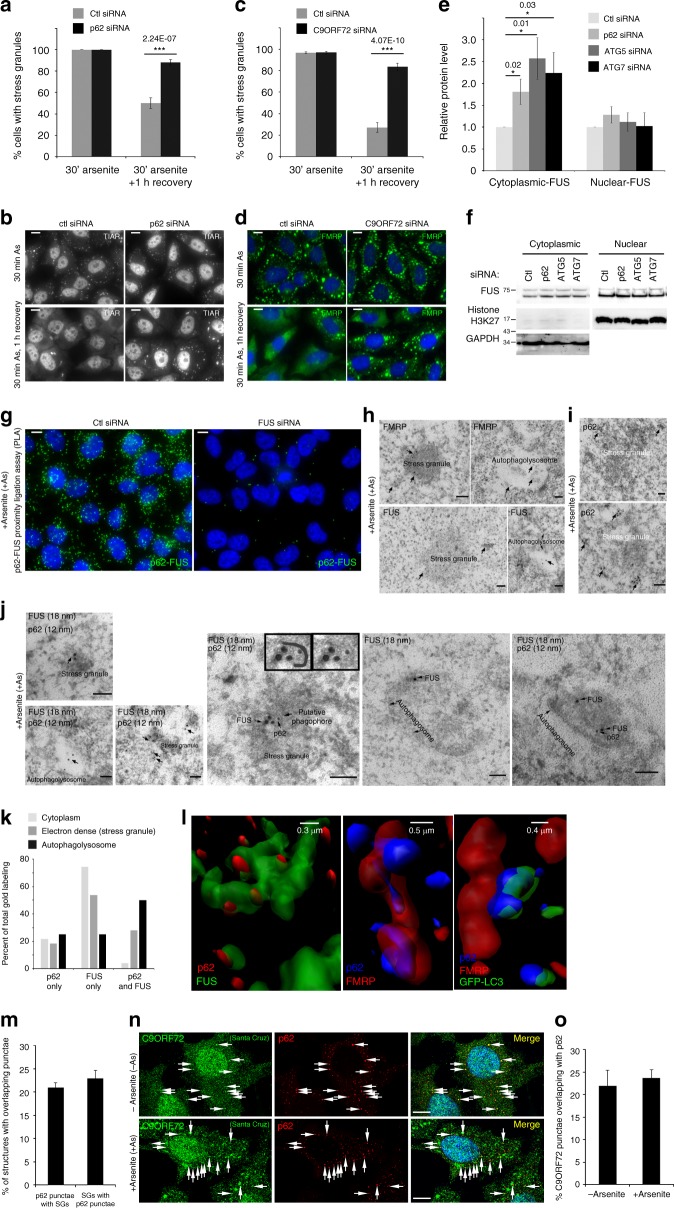
C9ORF72 and p62 promote stress granule clearance by autophagy. **a** Percentage of cells containing stress granules in cells treated with control or p62 siRNA after 30 min arsenite, or one hour after removal of arsenite (*n* = 3, mean ± SD, Student’s *t*-test). **b** Representative images of stress granules used in (**a**). **c** Percentage of cells containing stress granules in cells treated with control or C9ORF72 siRNA after 30 min arsenite, or one hour after removal of arsenite (*n* = 3, mean ± SD, Student’s *t*-test). **d** Representative images of stress granules used in (**c**). **e** Quantification of FUS levels in cytoplasmic and nuclear extracts of cells treated with siRNA targeting p62, ATG5, ATG7, or control siRNA (*n* = 5, mean ± SD, Student’s *t*-test). **f** Representative western blot of (**e**). **g** Proximity ligation assay for p62 and FUS in arsenite-treated cells transfected with control siRNA or siRNA targeting FUS. Scale bar = 10 µm. **h** Immuno-electron micrographs of FUS and FMRP in cells treated with arsenite; arrows highlight antibody labeling within indicated stress granule-like structures. **i** Immuno-electron micrographs of p62 in cells treated with arsenite. **j** Immuno-electron micrographs of p62 (12 nm gold) and FUS (18 nm gold) in cells treated with arsenite; arrows highlight co-labeled structures. Scale bar = 100 nm. **k** Quantification of 46 randomly selected immuno-electron micrographs co-labeled with p62 and FUS as in (**j**). Each electron-dense stress granule-like structure, autophagolysosome-like organelle or 50 nm radius area of cytoplasm with label was quantified as being labeled with p62 alone, FUS alone, or both p62 and FUS. **l** 3D reconstruction of Z-stack confocal images of cells. Images are zoomed-in on stress granules labeled with the indicated antibodies or fluorescent proteins (GFP-LC3). **m** Quantification of the percentage of p62 punctae co-labeled with stress granule markers and the percentage of stress granules with co-localized or docked p62 (quantified 12102 p62 punctae and 12627 stress granules, *n* = 6, mean ± SD, Student’s *t*-test). **n** Confocal image of endogenous C9ORF72 and p62 in cells treated with or without arsenite. Scale bar = 10 µm. **o** Quantification of the percentage of C9ORF72 punctae overlapping with p62 in cells treated with or without arsenite (quantified C9ORF72 punctae without arsenite: 2688, with arsenite: 3778 punctae; p62 punctae without arsenite: 1805, with arsenite: 2942, *n* = 2, mean ± SD, Student’s *t*-test). HeLa cells were used in all experiments

To test if C9ORF72 and p62 control elimination of stress granules, p62 or C9ORF72 were depleted with siRNA. Depletion of C9ORF72 had minimal effect on p62 levels (Supplementary Fig. 1b). Cells depleted of p62 or C9ORF72 formed similar numbers of stress granules (30 min arsenite) as control cells (Fig. 2a–d). In contrast, during recovery from stress (1 h recovery after 30 min arsenite), 88 or 84% of cells, respectively, treated with siRNA targeting *p62* (Fig. 2a, b) or *C9ORF72* (Fig. 2c, d) failed to eliminate stress granules, closely mimicking the effect of inhibiting autophagy with ATG5 siRNA (Supplementary Fig. 2a, b). p62 knockdown had no effect on stress granule numbers in *ATG5*^−/−^ cells suggesting that p62 operates in a ATG5-dependent autophagy pathway to clear stress granules (Supplementary Fig. 2c). In contrast, depletion of other autophagy receptors including NBR1 and OPTN did not affect clearance of stress granules (Supplementary Fig. 2d). Consistent with degradation of cytoplasmic stress granules by p62-dependent autophagy, cytoplasmic fractions, but not nuclear fractions, accumulated the stress granule protein FUS in cells treated with siRNA targeting p62, or genes required for autophagy (ATG5 and ATG7, Fig. 2e, f, Supplementary 2b).

If p62 directly affects clearance of stress granules (Fig. 2a, b, Supplementary 2a–c); it may localize to stress granules. P62 associated with the stress granule proteins FUS, SMN, and DDX3 by immunoprecipitation (Fig. 1m, n). PLA confirmed that p62 and FUS associate in intact cells (Fig. 2g). To test whether p62 physically associates with stress granules we used electron microscopy. As expected, p62-labeled mitochondria^24,25^, as well as endolysosomal structures with heterogenous content resembling autophagolysosomes (Supplementary Fig. 2e). In arsenite-treated cells, stress granules were observed, as previously described^26^, as electron-dense structures labeled with the stress granule markers FUS and FMRP (Fig. 2h). p62 frequently clustered on the periphery of stress granules (Fig. 2i). FUS and FMRP were also observed on similar electron-dense structures inside autophagolysosome-like structures (Fig. 2h), suggesting their degradation by autophagy. In agreement, p62 and FUS were infrequently found in close proximity in the cytoplasm (Fig. 2k) but 28% of structures co-labeled by p62 and FUS were stress granules and another 50% were inside autophagolysosomes (Fig. 2j, k). In agreement with the role of autophagy in engulfment of stress granules, p62-labeled phagophore-like structures also associated with stress granules (Fig. 2j), stress granules labeled with FUS and p62 were observed within double-membrane autophagosomes (Fig. 2j) and stress granule proteins TIAR and SMN accumulated in cells depleted of ATG5 (Supplementary Fig. 2f). Together, this suggests that either entire stress granules or fragments of stress granules are degraded by p62-dependent autophagy and this is required for complete elimination of stress granules from cells (Fig. 2a–f, Supplementary Fig. 2a–c).

Intriguingly, by electron microscopy p62 was frequently observed on the periphery of stress granules (Fig. 2i, j), as required to remain accessible to LC3 and direct their degradation by autophagy. In agreement with this, 3D reconstructions of confocal microscopy images demonstrated that p62 docked on stress granules and LC3 coated p62 on these stress granules (Fig. 2l, Supplementary Fig. 2g, h). By immunofluorescence, p62 docked on 21% of stress granules (Fig. 2m, Supplementary 2g, h), closely paralleling the percent of stress granules (28%) containing p62 by electron microscopy (Fig. 2j, k). Costes’ test demonstrated that co-localization of p62 with TIAR in the cytoplasm is not random (in each of 88 cells, 100 iterations of randomized fluorescence found no images with greater co-localization than input images of p62 and TIAR). This closely resembles the type and amount of localization of autophagy receptors or LC3 to other autophagy substrates^27–29^ including stress granules^7^. This amount of co-localization is also consistent with the fact that not all stress granules are degraded by autophagy^7^, p62 is involved in degradation of other substrates, and autophagic degradation is rapid (~10 min).

Since C9ORF72 and p62 form a complex (Fig. 1b, e–j) and control elimination of stress granules (Fig. 2a–d), they may dock on stress granules. Endogenous C9ORF72 and p62 co-localized both in untreated and arsenite-treated cells (Fig. 2n, o). PLA detected an association of C9ORF72 and p62 on the border of some stress granules (Supplementary Fig. 2i). Together, while C9ORF72 may also have effects on stress granules independent of p62, this suggests that C9ORF72 and p62 form a complex that docks on stress granules to control their elimination by autophagy either as large structures or portions of stress granules (Figs. 1, 2, Supplementary Figs. 1, 2).

### PRMT5 helps eliminate stress granules by autophagy

**Fig. 3:**
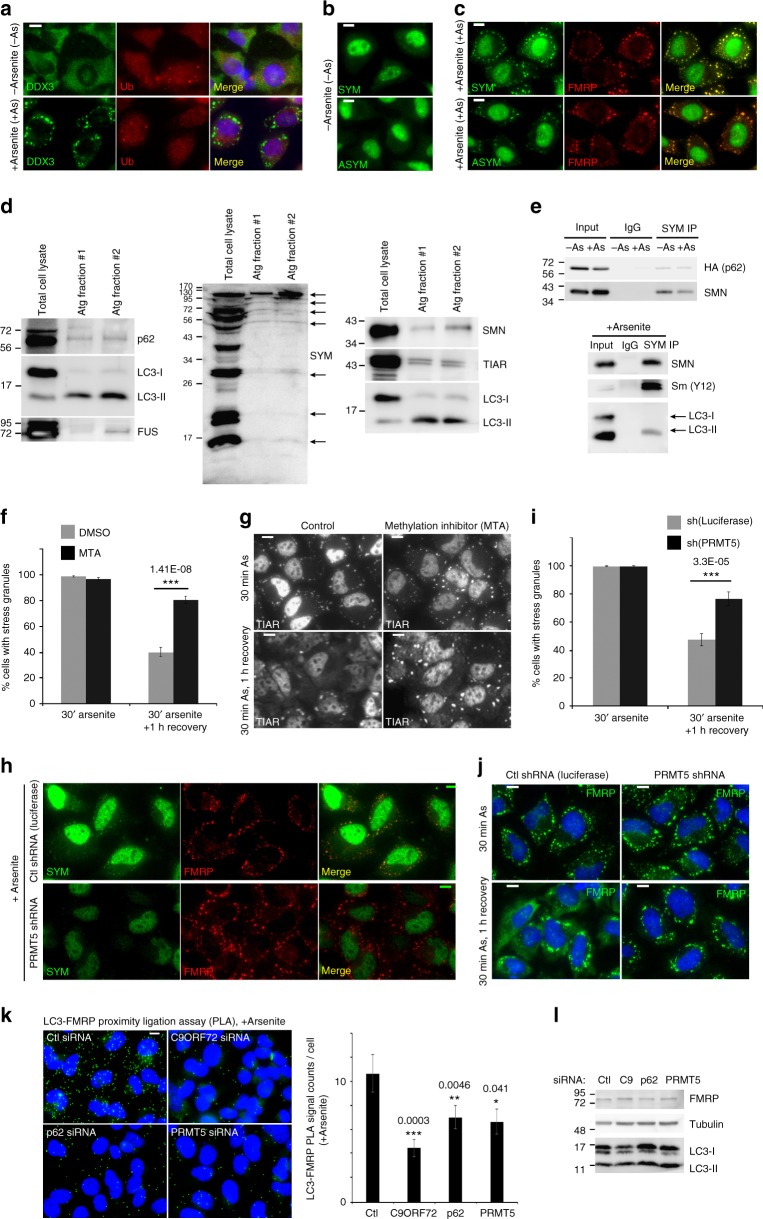
Symmetric arginine methylation by PRMT5 is required for stress granule clearance by autophagy. **a** Immunofluorescent microscopy of DDX3 and ubiquitin (Ub) in cells treated with or without arsenite. **b** Immunofluorescent microscopy of cells labeled with antibodies for symmetric (SYM) or asymmetric (ASYM) dimethylated arginines. **c** Immunofluorescent microscopy of cells labeled with antibodies for FMRP and proteins containing symmetric (SYM) or asymmetric (ASYM) arginines after treatment with arsenite. **d** Western blot of total cell lysate and fractions enriched in autophagosomes (Atg fraction#1) and autophagolysosomes (Atg fraction#2) for known autophagy substrates (LC3-II, p62), stress granule proteins (FUS, SMN, TIAR), and symmetrically dimethylated proteins (SYM). **e** Western blot of immunoprecipitations of proteins containing symmetrically dimethylated arginines from cells expressing HA-p62 (top) or not (bottom); cells were treated with arsenite (+As) or not (−As). **f** Percentage of cells containing stress granules in cells treated with DMSO (control diluent) or MTA (methylthioadenosine 1 mM, 48 h) after 30 min arsenite treatment, or one hour after removal of arsenite (*n* = 3, mean ± SD, Student’s *t*-test). **g** Representative images of stress granules labeled with TIAR for (**f**). **h** Immunofluorescent images of cells labeled with antibodies specific for symmetrically dimethylated arginines (SYM) or FMRP (stress granule marker) in cells treated with arsenite and expressing either control shRNA or PRMT5 silencing shRNA. **i** Percentage of cells containing stress granules in cells treated with control shRNA or PRMT5 silencing shRNA after 30 min arsenite treatment, or one hour after removal of arsenite (*n* = 5, mean ± SD, Student’s *t*-test). **j** Representative images of stress granules labeled with FMRP for (**i**). **k** Left, PLA for endogenous LC3 and FMRP (in SGs) in cells treated with arsenite and transfected with control (Ctl), C9ORF72 (C9), p62, or PRMT5 siRNA; Right, quantification of proximity ligation assay signals (PLA) for LC3 and FMRP in cells under identical conditions to (**k**) (*n* = 3, mean ± SD, Student’s *t*-test). **l** Western blot control for PLA in (**k**) in cells treated with control, C9ORF72, p62, or PRMT5 targeting siRNA. HeLa cells were used in all experiments. Scale bar = 10 µm

We inquired how a C9ORF72-p62 complex eliminates stress granules by autophagy. Canonically, p62 recruits ubiquitinated cargoes for degradation by autophagy^3^. Ubiquitin was not enriched in stress granules despite forming punctae in cells treated with inhibitors of autophagic degradation and co-localizing with OPTN, a ubiquitin-binding autophagy receptor (Fig. 3a, Supplementary Fig. 3a, b). This suggested that p62 may be recruited to stress granules by a non-canonical signal. Interestingly, arginine-dimethylated proteins, particularly symmetrically dimethylated proteins (SYM), were strongly enriched in stress granules (Fig. 3b, c). Asymmetrically dimethylated proteins (ASYM) were also found in stress granules (Fig. 3b, c). Subcellular fractions were prepared according to established protocols^30^ to enrich autophagosomes and autophagolysosomes. Accordingly, these fractions were enriched in LC3-II (vs. LC3-I) compared to the LC3-II/LC3-I ratio in the corresponding total cell lysates (Fig. 3d). Comparing the ratio of proteins in the autophagolysosome fractions to their level in the corresponding cell lysate indicates the propensity of proteins to be degraded by autophagy. Autophagolysosome fractions contained the canonical autophagy substrate p62, stress granule proteins degraded by autophagy such as FUS (Fig. 2e–m), and SYM at similar autophagosome fraction/cell ratios (Fig. 3d), suggesting that proteins with symmetrically dimethylated arginines are degraded at a rate comparable to p62 and stress granules. Additionally, SYM accumulated upon depletion of ATG7 and ATG12 (Supplementary Fig. 3c). p62 and LC3 also immunoprecipitated with proteins containing symmetrically dimethylated arginines (Fig. 3e), alongside proteins known to contain (Sm) or bind to (SMN) this post-translational modification (Fig. 3e). This suggests that proteins with symmetrically dimethylated arginines are enriched in stress granules, associate with p62 and are degraded by autophagy.

We tested whether symmetric arginine dimethylation is required for elimination of stress granules. The methylation inhibitor, methylthioadenosine (MTA) (Supplementary Fig. 3d) alone did not induce stress granules and had minor effects on cell survival (Supplementary Fig. 3e). In cells treated with arsenite, MTA did not affect stress granule formation but prevented their elimination (Fig. 3f, g). Depletion of the symmetric dimethylation enzyme PRMT5 (Fig. 3h, Supplementary Fig. 3f, h–i) did not affect autophagosome formation or p62 levels (Supplementary Fig. 3f, g). PRMT5 depletion abrogated symmetric arginine dimethylation in stress granules (Fig. 3h) and prevented their elimination during recovery from stress (Fig. 3i, j). A PRMT5 inhibitor (EPZ015666) had similar effects (Supplementary Fig. 3j-m). Cumulatively, this suggests that like C9ORF72 and p62, symmetric arginine dimethylation of stress granule proteins by PRMT5 is required for their elimination. Knockdown of C9ORF72, p62, or PRMT5 decreased recruitment of LC3 to the stress granule marker FMRP as measured by PLA (Fig. 3k, Supplementary 3n) independent of any change in LC3 or FMRP levels (Fig. 3l). With evidence that p62 is epistatic to ATG5 in eliminating stress granules (Supplementary Fig. 2a–c), and the presence of stress granule markers with p62 in autophagosomes and autophagolysosomes by density gradient (Fig. 3d), confocal microscopy (Fig. 2l), and electron microscopy (Fig. 2h–k), this strongly suggests that a complex of C9ORF72 and p62 (Fig. 1) requires PRMT5 to eliminate stress granules by autophagy.

### Methylation of FUS by PRMT5 is required for binding to p62

**Fig. 4:**
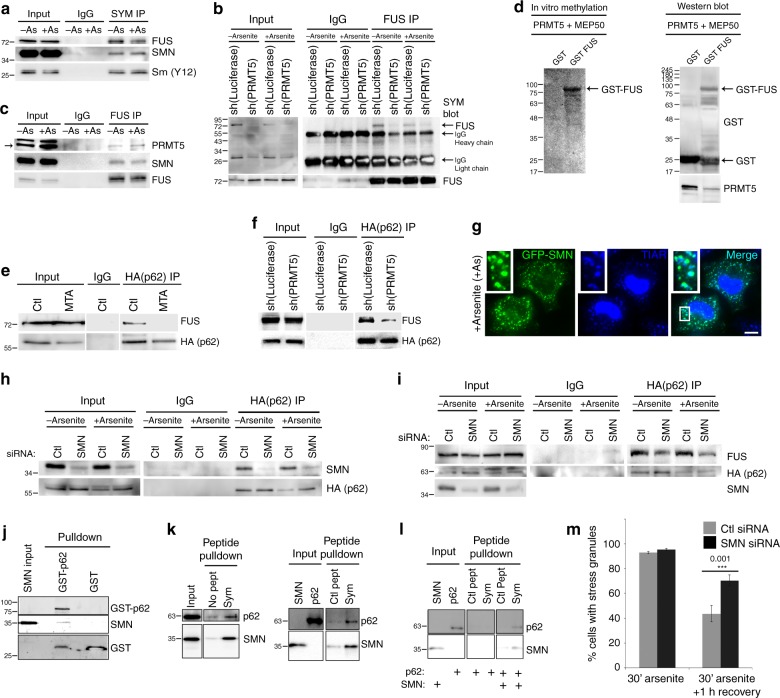
SMN mediates the interaction of p62 with FUS methylated by PRMT5 for degradation by autophagy. **a** Western blot of immunoprecipitates of endogenous proteins containing symmetrically dimethylated arginines from cells treated with arsenite (+As) or not (−As). **b** Western blot for proteins containing symmetrically dimethylated arginines in immunoprecipitates of FUS from cells stably expressing PRMT5 shRNA or control shRNA; top arrow indicates mass of FUS on western blots and bottom arrows indicate bands due to detection of immunoprecipitating IgG heavy and light chain. **c** Western blot of immunoprecipitates of FUS from cells with and without arsenite treatment (+As, −As); A non-specific band appears above PRMT5 in PRMT5 input western blots that is not depleted by shRNA targeting PRMT5 (see Supplementary Fig. 3e). **d** Left, autoradiograph of (3)H after in vitro methylation assay of GST or GST-FUS incubated with PRMT5-MEP50; Right, western blot of PRMT5 and GST in samples used for in vitro methylation assays (Right). **e** Western blot of HA-p62 immunoprecipitates in cells treated with DMSO (Ctl) or MTA (methylthioadenosine 1 mM, 48 h). **f** Western blot of FUS in HA-p62 immunoprecipitates from cells stably expressing PRMT5 or control shRNA. **g** Immunofluorescent microscopy of GFP-SMN and TIAR (stress granule marker) in cells treated with arsenite. **h** Western blot of SMN in immunoprecipitates of HA-p62 from cells treated with SMN or control siRNA and either treated or not with arsenite. **i** Western blot of FUS in immunoprecipitates of HA-p62 from cells treated with siRNA targeting SMN or control (Ctl) and either treated or not with arsenite. **j** Western blot of recombinant SMN and GST-p62 from in vitro GST-pulldown assay. **k** Western blot of recombinant p62 and SMN from in vitro pulldown of control (Ctl) or symmetrically dimethylated peptides (Sym) incubated with recombinant p62 and SMN. **l** Western blot of recombinant p62 and SMN from in vitro pulldown of peptides incubated with recombinant p62 alone or p62 and SMN. **m** Percentage of cells transfected with control (Ctl) or SMN siRNA containing stress granules following 30 min arsenite treatment or following 1 h recovery from arsenite (*n* = 6, mean ± SD, Student’s *t*-test). HeLa cells were used in all experiments. Scale bar = 10 µm

Candidate interactors of C9ORF72 and p62 included several proteins containing dimethylated arginines by mass spectrometry, including FUS (Supplementary Table 6). We asked whether FUS could be symmetrically dimethylated by PRMT5. PRMT1 is known to asymmetrically dimethylate arginines in FUS^31^, however FUS is not known to be symmetrically dimethylated, or dimethylated by PRMT5. FUS was detected in immunoprecipitates of proteins with symmetrically dimethylated arginines (Fig. 4a), and a protein at the mass of FUS was detected with antibodies specific for symmetrically dimethylated arginines in FUS immunoprecipitates (Fig. 4b). PRMT5 was detected in FUS immunoprecipitates along with SMN which binds SYM (Fig. 4c), suggesting that PRMT5 may symmetrically dimethylate FUS. In agreement, symmetric dimethylation of arginine residues on FUS was reduced upon PRMT5 depletion (Fig. 4b) and PRMT5 methylated GST-FUS, but not GST in an in vitro radiolabeling assay (Fig. 4d). This suggests that PRMT5 symmetrically dimethylates FUS in vitro and in vivo.

Intriguingly, FUS was only detected in p62 immunoprecipitates by mass spectrometry in its arginine dimethylated form (Supplementary Tables 5, 6). This suggests that dimethylation of FUS may be required for its binding to p62. In agreement, FUS immunoprecipitated with p62 and this association was strongly reduced in cells treated with the methylation inhibitor MTA or depleted of PRMT5 (Fig. 4e, f).

### The Tudor protein SMN mediates association of FUS and p62

We investigated how p62 associates with arginine-methylated FUS. Tudor domains bind dimethylated arginines^32^. Survival motor neuron protein (SMN) is a Tudor protein that preferentially binds symmetrically methylated arginines^33^, frequently co-localizes with or docks on stress granules like p62 (Fig. 4g)^34^, and associates with FUS^35^ (Fig. 4c, Supplementary Fig. 4a). Like FUS, SMN immunoprecipitated with p62 in the absence or presence of oxidative stress (Figs. 1n, 4h). This suggests that SMN may link FUS to p62. Supporting this mechanism, association of FUS with p62 was reduced when SMN was depleted with siRNA (Fig. 4i, Supplementary Fig. 4b).

We sought to investigate binding of p62 to symmetrically methylated arginines and SMN in more detail using recombinant proteins. Recombinant SMN was pulled down with recombinant GST-p62, but not GST alone (Fig. 4j). In agreement with direct binding of recombinant p62 and SMN, p62 binding to SMN in cells did not require the p62 ubiquitin-binding domain (UBA) or its LC3 interacting region (LIR) (Supplementary Fig. 4c). In subsequent assays, peptides containing symmetrically dimethylated peptides were biotin-labeled and used for pulldowns compared to no peptide or identical peptides lacking symmetric dimethylation. As expected^33^, SMN exhibited more binding to peptides containing symmetric arginine dimethylation (Fig. 4k). p62 exhibited some background binding to beads likely due to its tendency to oligomerize and aggregate, but in the presence of SMN, p62 bound more to beads containing symmetrically dimethylated peptides (Fig. 4k). This demonstrates that p62 can bind the Tudor protein SMN (Fig. 4j) and requires SMN to bind to peptides containing symmetric dimethylation (Fig. 4k, l). Replicating the phenotype of p62 and PRMT5 knockdown (Figs. 2a, b, 3i, j), depletion of SMN did not impede formation of stress granules but blocked their elimination during recovery from stress (Fig. 4m, Supplementary 4d). This suggests that p62 uses SMN to recognize arginine dimethylated proteins including FUS in stress granules for clearance by autophagy.

### PRMT5 methylates FUS on R218 for autophagic degradation

**Fig. 5:**
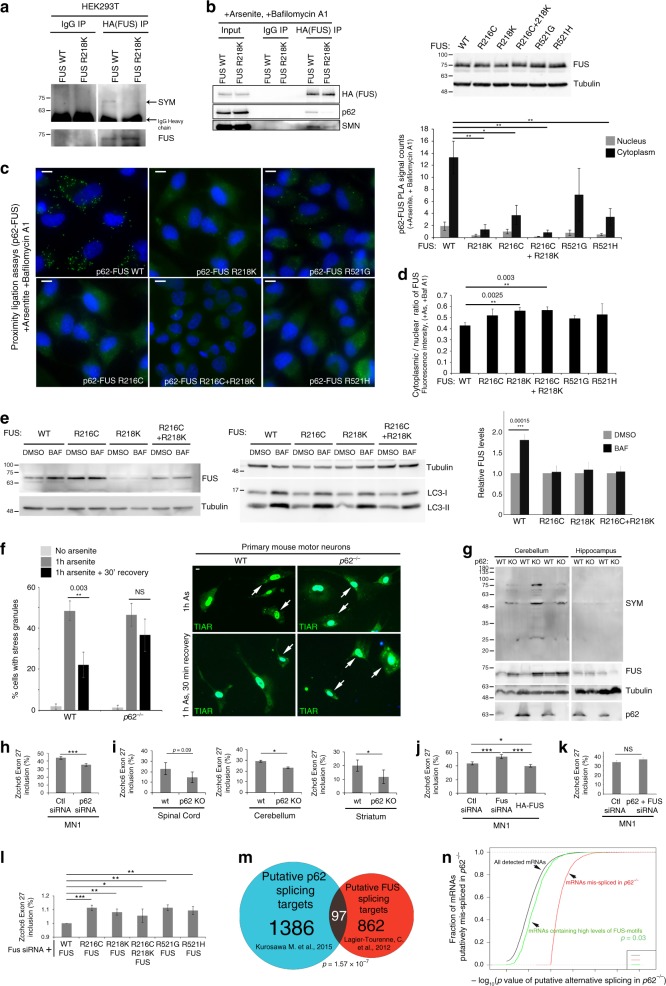
p62 regulates turnover of FUS and splicing of its targets. **a** Western blot of symmetrically dimethylated arginines in FUS immunoprecipitates from HEK293T cells. **b** Left, western blot of FUS immunoprecipitates from HeLa cells treated with arsenite and Bafilomycin A1; Right, western blot of FUS in HeLa cells in conditions used for PLA in (**c**). **c** Left, PLA of p62 and wild type or indicated FUS mutants in HeLa cells treated with arsenite and Bafilomycin A1; Right, quantification of PLA; images counted (>10 cells/image): wild type (11), R218K (9), R216C (8), R216C + R217K (8), R521G (9), R521H (9) (*n* = 3, mean ± SD, Student’s *t*-test). **d** Quantification of nuclear/cytoplasmic distribution of wild type and mutant FUS by fluorescence intensity in HeLa cells treated with arsenite and Bafilomycin A1; cells counted: wild type (32), R218K (19), R216C (8), R216C + R218K (16), R521G (31), R521H (21) (mean ± SD, Student’s *t*-test). **e** Left, western blot of FUS wild type, indicated mutants and LC3 upon Bafilomycin A1 treatment of HeLa cells; Right, quantification of western blots normalized to Tubulin (*n* = 6, mean ± SD, Student’s *t*-test). **f** Left, percentage of cells containing SGs in primary motor neuron cultures of wild type and *p62*^*−/−*^ mice (*n* = 2–4); 35–150 cells were counted per condition (mean ± SD, Student’s *t*-test); Right, representative images. **g** Western blot of lysates from wild type (WT) and *p62*^*−/−*^ (KO) mice. **h**–**l** RT-PCR analysis of Zcchc6 splice variants in (**h**) p62 depleted MN1 cells (*n* = 4), **i** indicated tissues of WT and *p62*^*−/−*^ mice (*n* = 3), **j** FUS depleted and overexpressing MN1 cells (*n* = 4), **k** FUS and p62 depleted MN1 cells (*n* = 3), and **l** MN1 cells depleted of endogenous FUS with siRNA targeting FUS 3’UTR and expressing FUS wild type or indicated mutants (*n* = 4, mean ± SEM, Student’s *t*-test). **m** Venn-diagram depicting overlap between mRNAs putatively alternatively spliced in *p62*^*-/-*^ brains and those bound to FUS and mis-spliced in *FUS*^*-/-*^ brains. **n** A plot of the fraction of mRNAs putatively mis-spliced in *p62*^*−/−*^ brain (*y*-axis) vs. the increasing significance of this (-log_10_ *p*-value, *x*-axis); black line—all detected mRNAs, green line—mRNAs enriched in FUS-binding motifs, red line—only mRNAs putatively mis-spliced in *p62*^*−/−*^ brain. *p* = 0.0357 that putative mRNAs mis-spliced in *p62*^*−/−*^ brains are enriched in FUS-binding motifs. Scale bar = 10 µm

PRMT5-mediated symmetric dimethylation promotes FUS association with p62 (Fig. 4a-f). FUS was previously observed to associate with symmetrically arginine-dimethylated proteins^36^, but whether and where it was directly symmetrically methylated remained unclear. FUS detected by mass spectrometry in both p62 and C9ORF72 immunoprecipitates was dimethylated exclusively on a peptide encompassing two arginines (R216, R218, Supplementary Table 6). Other dimethylation sites were identified; however methylation at R218 was the only site consistently identified in assays including p62 and C9ORF72 interactomes, increasing in methylation assays with PRMT5 (Fig. 4d, Supplementary Table 7), and decreasing in cells expressing PRMT5 shRNA (Supplementary Fig. 5a). In some cases, FUS peptides were fragmented between R216 and R218, and this confirmed FUS is dimethylated on R218 (Supplementary Fig. 5b). To confirm this, R218K mutation of FUS strongly decreased its symmetric arginine dimethylation detected by western blot (Fig. 5a, Supplementary Fig. 5c). This suggests that R218 is a principal site of PRMT5-dependent symmetric dimethylation on FUS.

PRMT5-dependent methylation of FUS promotes its binding to SMN and p62 (Fig. 4f–l). FUS R218K mutants and others were expressed at similar or slightly higher levels in cells (Fig. 5b, Supplementary Fig. 5d). In agreement with R218 of FUS being the principal site of symmetric dimethylation by PRMT5, the immunoprecipitation of p62 and SMN with FUS was reduced by mutation of R218 (Fig. 5b). In agreement, similar amounts of FUS R218K and other FUS mutants localized to the cytoplasm as wild-type FUS during oxidative stress (Fig. 5d, Supplementary Fig. 5e), but in PLA p62 association with FUS was ablated by the R218K mutation (Fig. 5c). Nonetheless, depletion of FUS with siRNA did not alter formation or elimination of stress granules (Supplementary Fig. 5f), suggesting that p62 recognizes multiple proteins methylated by PRMT5 to promote elimination of stress granules. If p62 can also eliminate symmetrically methylated proteins individually or in small clusters then mutating methylation sites should block their turnover by autophagy. Wild-type FUS significantly accumulated when autophagic degradation was blocked with Bafilomycin A1 whereas R218K mutant was unperturbed demonstrating that it is no longer targeted for degradation by autophagy (Fig. 5e, Supplementary Fig. 5g). This strongly suggests that symmetric arginine dimethylation of FUS at R218 by PRMT5 is required for its association with p62 and clearance by autophagy (Figs. 3i-k, 4, 5a-e), while methylation of multiple proteins in stress granules is likely required for their elimination by autophagy.

Like FUS R218K, the association of FUS R216C and R521H but not R521G with p62 was strongly decreased, despite accumulating similarly in the cytoplasm and being expressed at similar levels (Fig. 5b–d, Supplementary Fig. 5e). The association of FUS with p62 could be impaired at multiple levels. For example, PRMT5 or SMN may no longer be able to access R218, or the FUS-SMN complex could be otherwise altered to impair p62 binding. Symmetric dimethylation of FUS was not decreased in R216C or R521H mutants (Supplementary Fig. 5h). While FUS R216C association with SMN was not impaired, FUS R521H mutant associated poorly with SMN (Supplementary Fig. 5h), suggesting that the C-terminus of FUS may be required with R218 for optimal interaction with SMN. FUS R216C was also unperturbed upon inhibiting autophagic degradation (Fig. 5e). Together, this suggests that multiple mutations in FUS have an impaired association with p62 caused by defects at multiple levels in the pathway elucidated here and this can prevent p62-dependent autophagy.

### *p62*^*−/−*^ mice accumulate symmetrically methylated proteins

We assessed whether p62 may affect elimination of stress granules in cell types relevant to ALS. Elimination of arsenite-induced stress granules in motor neurons derived from *p62*^*−/−*^ mice was impaired compared to their wild-type counterparts (Fig. 5f). Both FUS and SMN immunoprecipitated with SYM in mouse cortex, cerebellum, and spinal cord (Supplementary Fig. 5i), suggesting that FUS is methylated by PRMT5 in relevant mouse tissues. SYM and FUS accumulated in cerebellum, but not the hippocampus of *p62*^*−/−*^ mice (Fig. 5g). While we cannot exclude that p62 promotes symmetric arginine dimethylation, this data suggests that p62 controls turnover of stress granules in neurons and proteins containing symmetrically dimethylated arginines in some regions of the mouse brain.

### *p62*^*−/−*^ mice exhibit FUS-dependent mRNA splicing defects

P62 may impact the amount and nuclear-cytoplasmic distribution of FUS and other splicing regulators that are symmetrically dimethylated resulting in alternative splicing. In brain of *p62*^*−/−*^ mice^37^, while no mRNAs besides p62 exhibited changes in expression, 1386 mRNAs exhibited signatures of alternative splicing. A predicted p62-dependent splicing event in *Zcchc6* (exon 27 inclusion) was impaired in p62 knockdown MN-1 cells and in several brain regions of *p62*^*−/−*^ mice (Fig. 5h, i, Supplementary Fig. 5j–l). Zcchc6 uridylates the 3′ end of select miRNA and mRNAs including *let-7*^38^ whereby it may impact formation of neuromuscular junctions^39^ and ALS-associated phenotypes^40^. Exon 27 inclusion of *Zcchc6* mRNA was increased when FUS was depleted with siRNA, reversed when wild-type FUS, but not FUS mutants (R218K, R216C, R521G, R521H) were expressed and further enhanced by blocking autophagic degradation of FUS with Bafilomycin (Fig. 5j, l, Supplementary Fig. 5m–o). The decreased inclusion of Zcchc6 exon 27 induced by loss of p62 (Fig. 5h, i) was prevented by depletion of FUS (Fig. 5k), demonstrating that p62 effects on splicing of *Zcchc6* require FUS. Overall, this strongly suggests that p62 and FUS regulate shared alternative splicing events that depend in part on methylation of R218 on FUS and its ability to associate with p62 and be degraded by autophagy (Fig. 5a–l, Supplementary Fig. 5a–o).

More broadly, mRNAs putatively mis-spliced in *p62*^*−/−*^ brains included a highly significant proportion of mRNAs (97 of 862, *p* = 1.57 × 10^−7^) that bound to FUS and were mis-spliced in FUS-deficient brains^41^ (Fig. 5m, Supplementary Fig. 5p). Furthermore, mRNAs mis-spliced in *p62*^*−/−*^ brain were significantly enriched in a FUS-binding motif^41^ (Fig. 5n). This suggests that p62 and FUS regulate alternative splicing of a large shared pool of transcripts.

### Patient inclusions contain arginine-methylated proteins

C9ORF72 associates with p62 and moderately lowering the levels of C9ORF72 prevents clearance of stress granules (Figs. 1b, e–j, 2a–d). ALS patients with C9ORF72 repeat expansions have reduced levels of C9ORF72^17^ and may therefore exhibit signatures of impaired p62-dependent clearance of proteins with symmetrically methylated arginines.

**Fig. 6:**
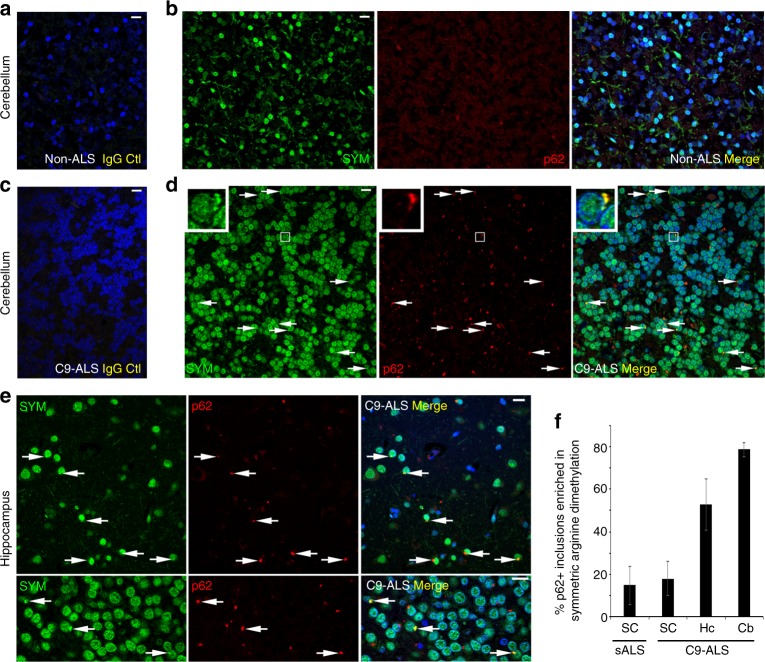
Patients with C9ROF72 repeat expansions exhibit inclusions rich in symmetrically arginine-dimethylated proteins. **a**, **b** Immunofluorescent microscopy of non-ALS human cerebellum labeled with (**a**) control non-specific antibodies or (**b**) antibodies specific for p62 and symmetrically dimethylated arginines. **c**, **d** Immunofluorescent microscopy of cerebellum of patient with C9ORF72 repeat expansions labeled with (**c**) control non-specific antibodies or (**d**) antibodies specific for p62 and symmetrically dimethylated arginines; arrows highlight inclusions containing p62 and symmetrically dimethylated arginines. **e** Immunofluorescent microscopy of hippocampus of patient with C9ORF72 repeat expansions labeled with antibodies specific for p62 and symmetrically dimethylated arginines; arrows highlight inclusions containing p62 and symmetrically dimethylated arginines. **f** Quantification of fraction of p62 inclusions positive for symmetrically dimethylated arginine proteins as observed in spinal cords (SC), hippocampi (Hc) and cerebellums (Cb) of patients with C9ORF72 repeat expansions (C9-ALS) and sporadic ALS (sALS). Scale bar = 10 µm. sALS Spinal Cord (*n* = 2); C9 ALS Spinal Cord (*n* = 2); C9 Hippocampus (*n* = 1); C9 Cerebellum (*n* = 3), mean ± SD, Student’s *t*-test. Cytoplasmic p62 inclusions were counted across 3–10 frames (10–80 p62 inclusions) in each sample

In patients with C9ORF72 expansions, inclusions were observed in the lumbar spinal cord containing TDP-43 and p62 as expected (Supplementary Fig. 6a, b). Validating the specificity of antibodies recognizing symmetrically dimethylated arginines, staining was reduced in PRMT5-depleted cells (Supplementary Fig. 3h), and exhibited prominent staining of the nucleus, without evident cytoplasmic foci in non-ALS patients as expected (Fig. 6a, b). In both cerebellum and hippocampus, but not spinal cord of patients with *C9ORF72* repeat expansions inclusions containing p62 frequently also contained proteins with symmetrically dimethylated arginines (Fig. 6c–f, Supplementary Fig. 6b). While we cannot exclude that p62 promotes symmetric dimethylation of arginines of inclusions, with evidence above, this suggests that p62 is recruited to inclusions containing symmetrically dimethylated arginines in patients with *C9ORF72* repeat expansions, but is unable to efficiently eliminate them, potentially due of the loss of C9ORF72 (Fig. 2c, d).

**Fig. 7:**
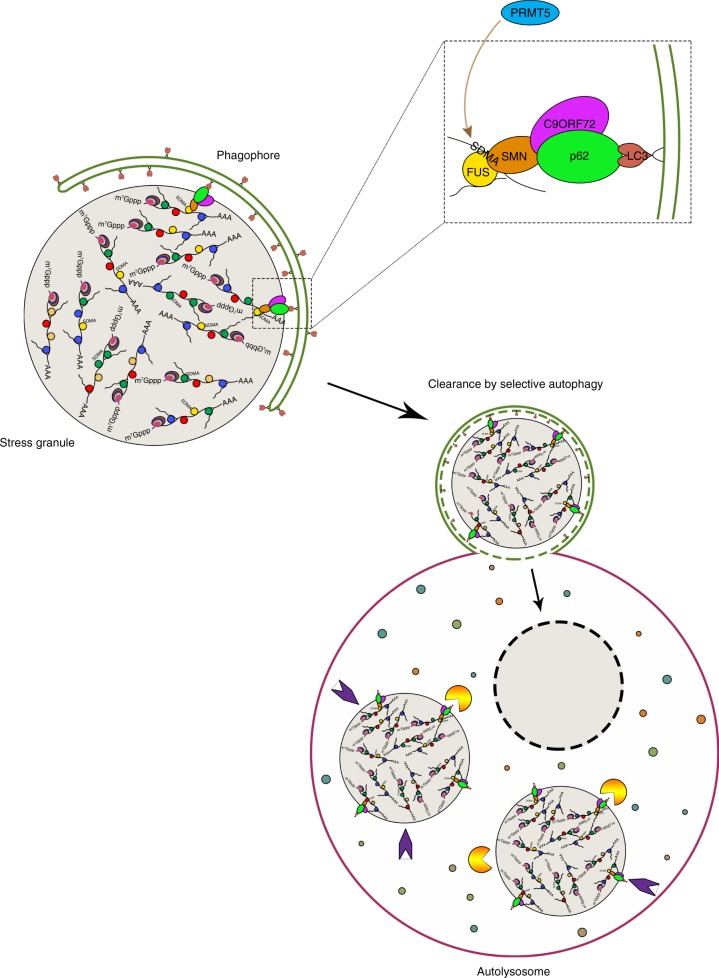
**Model**. Stress granule proteins such as FUS are symmetrically dimethylated on arginines by PRMT5 to bind Tudor-domain containing protein SMN for subsequent recruitment and degradation by p62-C9ORF72 mediated selective autophagy

Together, this suggests that C9ORF72 and p62 form a complex that recognizes proteins like FUS containing symmetrically dimethylated arginines to eliminate them individually or as clusters in stress granules by autophagy (Fig. 7). Interruption of this process can cause accumulation of symmetrically methylated proteins as demonstrated in the mouse brain and in the cytoplasm of patients with C9ORF72 repeat expansions.

## Discussion

Canonically, autophagy receptors bind ubiquitin on cargoes to direct their degradation by selective autophagy^42^, but this is not always the case^4^. While ubiquitin was not enriched in stress granules in our analyses, we cannot exclude that elimination of stress granules requires ubiquitination in addition to symmetric arginine methylation. Recent reports highlight the ability of alternative post-translational modifications to target cargoes for autophagic degradation. For example, P granules, an RNA-containing structure unique to *C.elegans* germline cells, are eliminated by autophagy^43^. Notably, this process is distinct from that described here in that it requires asymmetric, not symmetric arginine methylation of P granule proteins, and is independent of the *C. elegans* p62 homolog^8^. Arginine-methylated proteins are enriched in RNA-binding domains^36,44^. In the absence of an enzyme known to remove arginine methylation from proteins in cells, autophagy is one mechanism that enables turnover of arginine methylated proteins regrouped in diverse RNA granules.

PRMT1 asymmetrically methylates arginines on FUS^31^ causing FUS mutants associated with ALS to accumulate in the cytoplasm in stress granules^45^. Similarly, PRMT1-mediated methylation of FMRP and other proteins drives their recruitment to stress granules^46^. The current data suggest that an independent type of arginine methylation by PRMT5 at FUS R218 controls a subsequent step, the targeting of cytoplasmic FUS for degradation by autophagy.

ALS-linked C9ORF72 with repeat expansions that decrease levels of endogenous C9ORF72 were previously shown to increase stress granule number, but the mechanism remained unknown^47^. Mimicking these conditions with a partial knockdown of C9ORF72, we observed a defect in elimination of stress granules (Fig. 2c, d). C9ORF72 also has a role in controlling the initiation of phagophore formation by interacting with Rab1a^48^. Intriguingly, Rab1a was identified in stress granules^22^. We found that C9ORF72 and p62 decorated stress granules to initiate their elimination by autophagy. This suggests C9ORF72 and p62 are part of a broader complex, potentially including Rab1a at stress granules to promote engulfment of stress granules or fragments thereof within autophagosomes. At the same time, we cannot exclude that C9ORF72 effects independent of p62, or p62 functions independent of autophagy also promote elimination of stress granules.

Electron microscopy exhibited p62 and FUS co-localized on small electron-dense clusters (~20–80 nm) on the periphery of stress granules (Fig. 2h–k) and p62 is required to eliminate stress granules (Fig. 2a, b, h–m). It is possible that p62 and autophagy achieve these effects by repeatedly eliminating parts of stress granules, rather than engulfing and degrading entire stress granules in a single autophagosome. Indeed, p62 bound to SMN and FUS both in the presence and absence of oxidative stress when stress granules are rare (Figs. 1n, 4h, i). Additionally, while methylation of FUS alone is insufficient to direct degradation of stress granules (Supplementary Fig. 5f), it is sufficient to cause degradation of FUS by autophagy (Fig. 5e). Together, this suggests that turnover of submicroscopic complexes of FUS and possibly individual FUS proteins may constitutively occur by p62-dependent autophagy and the clustering of multiple SYM into large granules during stress renders this process more readily observable with a microscope.

Multiple RNA surveillance mechanisms patrol the cytoplasm to prevent translation of mRNAs that contain errors or are unspliced^49,50^. Symmetrically methylated proteins are highly enriched in the nucleus and many are involved in RNA processing. Our data suggests that symmetrically methylated proteins that escape into the cytoplasm are targeted for degradation by p62-dependent autophagy. The mechanism we describe may be a constitutive RNA surveillance and quality control mechanism that detects escape of nuclear RNA processing complexes into the cytoplasm and restricts production of toxic proteins from immature mRNA or mRNA with errors.

One model suggests that stress granules or a related derivative underlie the pathology of at least some forms of ALS^13^. Mutations in another protein genetically linked to ALS, VCP, inhibit its normal function in promoting elimination of stress granules by autophagy^7^. Our data demonstrates a cascade of events governing the recognition and degradation of stress granules that requires a protein genetically linked to ALS at each step (FUS, SMN, p62, C9ORF72) (Fig. 7). This reinforces a unifying model in which mutations that instigate cytoplasmic accumulation of RNA splicing proteins or impinge on their elimination by autophagy can culminate in ALS.

Interestingly, multiple proteins modified with dimethyl-arginines associated with both C9ORF72 and p62. Proteins bearing these dimethyl sites included several proteins genetically linked to ALS, such as FUS, HNRNPA1, and TAF15 (Supplementary Table 6). This suggests that arginine dimethylation of these multiple ALS-linked substrates may drive association with p62 and elimination of stress granules. As we describe for ALS-linked FUS mutants (Fig. 5a–c, e), it is possible that some ALS-linked mutations in these other proteins disrupt the ability of p62 to recognize and eliminate stress granule-linked substrates by autophagy.

Repeat expansions in *C9ORF72* are the most common genetic cause of ALS^15,16^. Overexpression of dipeptide repeats translated from these repeat expansions causes cytoplasmic accumulation of RNA-binding proteins usually concentrated in the nucleus and can initiate the formation of inclusions^51,52^. However, repeat overexpression does not replicate all facets of the spinal cord motor neuron pathology and other symptoms observed in ALS patients^53^. Cells depleted of C9ORF72 to mimic reduced C9ORF72 expression in patients^17^ were incapable of targeting stress granules for p62-dependent autophagy (Figs. 1, 2). In ALS patients with C9ORF72 repeat expansions symmetrically arginine-methylated proteins accumulate in cytoplasmic foci often co-labeled with p62 (Fig. 6d–f).

Our evidence, thus suggests that loss of C9ORF72 may be required to generate the complete spectrum of ALS pathology, by preventing stress granule components from being eliminated and thereby contributing to inclusion formation, misregulation of splicing and attendant pathology in these ALS patients.

## Materials and methods

### Animal studies

*p62 WT and p62*^−/−^ mice were a gift from Dr. Herbert W. Virgin (Washington University—School of Medicine)^54^. Animal husbandry was performed in accordance with federal guidelines and the University of Ottawa Animal Care Committee. Information of *p62 WT* and *p62*^*−/−*^ mice used in the study is included in Supplementary Table 8.

### Isolation of mixed motor neurons

Mice embryos were collected from pregnant mice between day E13.5 and E14.5. Spinal cords and cortices were dissected using Zeiss Stereo Discovery V20 microscope (Carl Zeiss, Oberkochen, Germany). Clean spinal cords were then placed in dissection buffer (sucrose 40 g/L, dextrose 1 g/L, and HEPES 2.4 g/L in PBS), minced with scissors and incubated with trypsin (Sigma-Aldrich) for 30 min at 37 °C. The cells were separated using a 1 mL pipette and placed in S.C. NFeed (MEM/HBSS, Hyclone, hormones (insulin 10 μg/mL, transferrin 200 μg/mL, BSA 10 μg/mL, putrescine 32 μg/mL, selenium 26 ng/mL, T3 20 ng/mL, hydrocortisone 9.1 ng/mL, progesterone 13 ng/mL, and NGF 5 ng/mL) Sigma-Aldrich, Horse serum 1.3%, Multicell and ABAM 1%, Multicell) neuronal culture media. The cells were then counted and seeded at 300,000 cells/well in 12-well plates pre-treated with poly-D-Lysine 1 mg/mL (Sigma-Aldrich) and Matrigel 0.5% (S.C., Corning, VWR). After four to seven days, mitotic cells were killed using Arabinofuranosylcytosine 1.4 μg/mL (S.C., Calbiochem).

### Cell culture, transfections, and treatments

HeLa (CCL2, ATCC) and MDA-MB-231 (HTB-26, ATCC) were cultured in Dulbecco’s Modified Eagle’s Medium containing 10% fetal bovine serum, 2 mM L-glutamine and penicillin-streptomycin. MDA-MB-231 (HTB-26, ATCC) cells were genetically deleted of ATG5 using CompoZr Zinc Fingers technology (Sigma-Aldrich). Motor-neuron-derived MN-1 cells were cultured in DMEM (GIBCO) supplemented with 10% fetal bovine serum. All stable lines were maintained in 2 μg/ml of puromycin.

For transient transfection with DNA, Lipofectamine2000 (11668-019, Invitrogen) was used. Silencer Select siRNAs (Life Technologies) were transfected at 10 nM concentration with RNAiMax (13778150, Invitrogen). The cells were harvested for analyses 48 h post transfection. siRNAs and plasmids used in the study are listed in Supplementary Tables 9, 10.

For all stress granule experiments, HeLa and MDA-MB-231 cells were induced with 0.5 mM sodium arsenite (71287, Sigma-Aldrich) for 30 min followed by a PBS wash and recovery in complete DMEM up to 1 h. Primary cultures of mixed motor neurons were induced with 0.5 mM sodium arsenite (71287, Sigma-Aldrich) for 1 h followed by a PBS wash and recovery up to 30 min. At least 100 cells were counted per experiment for quantification of cells containing stress granules. HeLa and MN-1 cells were treated with Bafilomycin A1 (196000, EMN Millipore) for 16 h at 400 nM. HeLa cells were treated with (i) Chloroquine (sc-205629A, Santa Cruz Biotechnology) for 16 h at 20 μM, (ii) MTA (Sigma-Aldrich) for 48 h at 1 mM, (iii) PRMT5 inhibitor—EPZ015666 (SML1421, Sigma-Aldrich) for 48 h at 3 μM, and (iv) Ubiquitin E1 inhibitor—PYR-41 (N2915, Sigma-Aldrich) for 3 h at 50 μM.

### Lentiviral vector production and transduction

HeLa cells stably expressing shRNA targeting PRMT5 (sh(PRMT5)) or luciferase (sh(luciferase)) were generated via lentiviral transduction. Lentivirus was produced through transfection of 293T cells with the packaging plasmid pLKO.1-sh. The media was harvested 48 h post transfection and filtered through a 0.45 μM PES membrane. HeLa cells were seeded in 10 cm plates (250,000 cells) 24 h before infection. The cells were infected with the lentivirus containing media along with 8 μg/mL of polybrene. After 48 h, media was changed and cells were selected with puromycin (2 μg/mL). Equivalent amounts of virions encoding shRNA targeting luciferase as a control was used to transduce HeLa cells. Knockdown in stable lines was validated by RT-PCR and immunoblotting.

### DNA constructions

The plasmid pDEST-FUS-WT was obtained from Addgene (#26374). Three different arginine (R) mutations were generated: (i) arginine to cysteine at position 216 (R216C), (ii) arginine to lysine at position 218 (R218K), and (iii) both mutations (R216C and R218K) by PCR using oligos indicated in the primers table. PCR reactions were digested with DPNI for 1 h and transformed in DH5α. DNA was purified from colonies and sent for sequencing. Constructs that were successfully mutated were then purified by MaxiPrep (Qiagen) as per manufacturer’s guidelines.

### Reverse transcription (RT)-PCR and quantitative RT-PCR

After RNA extraction with Trizol, RT-qPCR was performed with Superscript II Reverse Transcriptase (Invitrogen) and GoTaq qPCR Master Mix (Promega, A6002). Fold change of transcripts was calculated using the ΔΔCt method, normalized to 18SrRNA. Sequences of primer pairs used in the study are listed in Supplementary Table 11.

For RT-PCR, cDNA was synthesized with random hexamers and Promega AMV cDNA synthesis (Promega, M5101). PCRs were then set up using Promega GoTaq DNA Polymerase (M5101) according to manufacturer’s instructions. RT-PCR conditions were as follows for validation of splicing targets: 95 °C for 2 min, (95 °C for 30 s, 55 °C for 30 s, and 72 °C for 45 s) × 32 cycles, and 72 °C for 10 min. Specific RT-PCR conditions used for particular primers are available upon request. Amplicons were run on a 2% agarose gel (containing 5 μL of EtBr 20 mg/mL) and visualized under UV light. For analysis of splice variants, densitometry was performed to measure percentage exon inclusion with or without normalization to wild-type condition.

### Isolation of autophagosomal fractions

Autophagosomes were fractionated as described^30^. Briefly, 20 160 cm^2^ plates of HEK293 cells were harvested in 0.25 M sucrose 10 mM HEPES pH 7.2, washed several times in the same solution, transferred into 0.25 M sucrose containing protease inhibitor cocktail (Roche, EDTA-free), and disrupted by nitrogen cavitation (Parr Instruments, 100 p.s.i. 30 s, 50 p.s.i. 30 s, and 25 p.s.i. 8 min). Lysate was centrifuged twice at 2000× *g* to eliminate intact cells and large debris. Supernatant was then centrifuged at 17,000× *g* (12 min) to pellet organelles. The pellet was resuspended in 0.25 M sucrose 10 mM HEPES, pH 7.2, and treated with a mixture of RNAse A/T1 (Fermentas) for 10 min, 37 °C. Organelles were then loaded at the bottom of a discontinuous Histodenz density gradient (26, 24, 20, and 15%) and centrifuged at 90,000× *g* for 3 h in an SW41 rotor (Beckman). Fractions at the interface of 15–20% and 20–24% densities are enriched in autophagosomes and autophagolysosomes, respectively. Autophagosome-enriched fractions were used for all analyses.

### Immunofluorescence and proximity ligation assay

Five micron sections were cut from formalin-fixed, paraffin-embedded blocks of cerebellum and hippocampus and mounted onto coated glass slides. Paraffin-embedded sections were deparaffinized in xylene (3 × 5 min) and rehydrated through graded EtOH to deionized water: 100% EtOH (2 × 5 min), 95% EtOH (2 × 5 min), 80, 70, and 50% EtOH (1 × 5 min). Additionally, frozen tissues were sectioned and mounted onto coated glass slides prior to fixation in 4% PFA in PBS (10 min). Antigen retrieval was performed in 0.05% Tween 20, 10 mM sodium citrate buffer pH 6.0 (prepared with 0.1 M citric acid) using a decloaking chamber (Biocare Medical, Walnut Creek, CA,USA) at 125 °C for 30 s and 90 °C for 10 s. The sections were cooled to room temperature and rinsed with deionized water five times and PBS once. They were permeabilized in 0.2% Triton-X-100 in PBS for 45 min, washed three times in PBS, blocked in 10% normal goat serum (S-1000, Vector Laboratories) in PBS for 1 h, and incubated with primary antibodies in blocking solution overnight at 4 °C. The sections were then washed thrice in PBS, incubated in fluorescently labeled secondary antibodies (1/250 dilution in PBS for 1 h), washed thrice in PBS, incubated in 0.5 μg/ml DAPI (D9542, Sigma-Aldrich) in PBS for 10 min, washed twice in PBS, and mounted with ProLong Diamond Antifade (P36961, Invitrogen).

Cells were prepared for microscopy as previously described^55,56^. The cells were grown on 0.17 mm glass coverslips prior to fixation in 4% PFA in PBS (10 min). The cells were rinsed in PBS and incubated 10 min in 0.2% Triton-X-100 in PBS containing 20 mM NH_4_Cl. After washing with PBS, the cells were blocked in 5% milk in PBS for 1 h, washed in PBS, and incubated with 1/200 dilution of primary antibody or control non-specific antibody overnight at 4 °C. For PLA, the cells were subsequently processed as per manufacturer’s instructions (DUO82049, Sigma-Aldrich). For immunofluorescence, the cells were subsequently washed thrice in PBS and incubated with 1/300 dilution of secondary antibodies (goat anti-mouse Alexa Fluor 488 or 546, anti-rabbit Alexa Fluor 488 or 546 or donkey anti-goat Alexa Fluor 488 (Invitrogen)) for 1 h. After three PBS washes, cells were mounted with Vectashield media (H-1200, Vector Laboratories) and imaged on a Zeiss AxioObserver Z1 epifluorescent microscope, a Zeiss 510 confocal microscope or a Zeiss LSM880 AxioObserver Z1 confocal microscope. At least three images were taken for each experiment.

### Quantification of cytoplasmic inclusions in ALS patients

P62 inclusions in the cytoplasm were manually counted across 3–10 frames of images per n. About 10–80 P62 inclusions were counted per n. The fraction of these P62 inclusions positive for symmetric arginine dimethylation was then manually ascertained.

### Quantification of microscopic data

Immunofluorescent images of punctae or stress granules containing P62, C9ORF72, and TIAR were first segmented using SQUASSH^57^. Segmented objects were then binarized prior to counting them using ‘Analyze Particles’ function of Fiji. Binarized images of objects from one channel were intersected with respective binarized objects from the other channel using ‘AND’ function of Fiji. The resulting number of overlapping punctae were then used to calculate % overlap. PLA dots were quantified using Duolink® ImageTool (DUO90806, Sigma-Aldrich).

### 3D reconstruction of Z-stacks

Z-stack images separated by 0.2 μm were acquired on a Zeiss LSM880 AxioObserver Z1 confocal microscope with Airyscan. 3D surfaces were rendered from Z-stack image files using Imaris.

### Electron microscopy

For transmission electron microscopy, cells were fixed in 2.5% glutaraldehyde in a 0.1 M sodium cacodylate buffer and then incubated in OsO4 (2%) in a 0.1 M sodium cacodylate buffer. After washing in 0.1 M sodium cacodylate the cells were dehydrated in increasing concentrations of alcohol. The most concentrated alcohol was replaced with acetone. The material was permeabilized with increasing concentrations of Araldite diluted in acetone. Finally, the tissues were embedded in Araldite (Huntsman Advanced Materials LLC, United States). Ultrathin sections (80 nm) were prepared using an Ultracut Leica UC6 ultramicrotome (Leica Microsystems, Germany) and placed onto copper grids coated with Formvar film. Sections were stained with uranyl acetate and lead citrate solutions and examined with a transmission electron microscope (JEOL JEM 1230, Japan).

For immungold labeling, the cells were fixed in 4% paraformaldehyde and 0.5% glutaraldehyde in 0.1 M sodium cacodylate buffer (pH 7.4). After dehydrating the samples with increasing concentrations of ethanol, the pellets were embedded in LR White Resin. The blocks were sectioned with ultracut (Leica EM UC 6) using a diamond knife. The thin sections were mounted on Formvar-coated nickel grids. The grids were floated on drops of 1% BSA/0.01% Tween 20 for 1 h, then incubated for 1 h with primary antibody or a mixture of rabbit and mouse primary antibody diluted with PBS-0.05% Tween 20 (PBST) diluted 1/200. Next, the samples were washed in PBST and incubated for 2 h with a mixture of secondary antibody in PBST. The secondary antibodies were 12-nm colloidal gold-conjugated goat anti-rabbit IgG antibody (111-205-144, Jackson) and 18-nm colloidal gold-conjugated goat anti-mouse IgG antibody (115-215-068, Jackson) that were applied at a dilution of 1/50 for 2 h at room temperature. For control staining the primary antibody was omitted and secondary only was used. After antibody labeling, the grids were washed three times in PBST, rinsed in distilled water, stained with uranyl acetate and lead citrate, and imaged with a transmission electron microscope (JEOL JEM 1230, Japan).

### Quantification of electron microscopy

Forty-six electron microscopy images containing any signal for P62 or FUS were collected at random from the cytoplasm of cells and 193 electron-dense clusters, autophagolysosomes or cytoplasmic labels were counted. P62 and FUS staining was evaluated as being associated with electron-dense stress granule-like structures whenever it or they were directly localized within a cluster of electron-dense material of at least 100 nm resembling structures labeled in other cells with stress granule markers FUS and FMRP. P62 and FUS was considered as within autophagolysosomes if it was within a translucent membrane-bound structure of >200 nm containing heterogenous contents. If more than one gold bead for P62 or FUS was associated with a single structure it was counted as a single co-localization event. P62 and FUS not localized to electron-dense clusters or membrane-bound organelles was labeled as cytoplasmic. If P62 and FUS were within 100 nm of each other in the cytoplasm they were considered co-localized.

### Co-immunoprecipitation

Cells were washed in cold PBS and lysed in NP40 lysis buffer (50 mM Tris-HCl pH 7.5, 150 mM NaCl, 1% NP40, Roche Complete Protease Inhibitor Cocktail Tablet EDTA free) for 30 min at 4 °C and centrifuged at 1000× *g*, 5 min prior to immunoprecipitation. Mouse brain and spinal cord tissues were homogenized in NP40 lysis buffer using Dounce homogenizer and centrifuged at 6000× *g*, 10 min at 4 °C. For IP, 500 μg lysate was pre-cleared with 5 μl (0.15 mg) protein G-Dynabeads (10004D, Life Technologies) for 20 min at 4 °C and estimated for protein content (DC Protein Assay, Bio-Rad). Every 500 μg lysate was incubated with 1 μg antibody for 3 h at 4 °C before incubation with 20 μl wet volume of protein G-Dynabeads (1.5 h, 4 °C). Alternatively, lysates with GFP or Myc-tagged proteins were directly incubated with 20 μl GFP-Trap®_A slurry (gta-20, ChromoTek) or 50 μl wet volume of anti-c-myc magnetic beads (PI88842, Thermo Fisher Scientific) for 2 h at 4 °C. Beads were collected by centrifugation or using a magnetic support (12321D, Thermo Fisher Scientific) and washed with 200 μl lysis buffer three times. SDS-sample buffer was added and the samples were boiled at 99 °C for 5 min prior to western blot analysis.

For RNAse treatments, equal amounts of lysate were untreated or treated with RNase A/T1 Mix (FEREN0551, Fermentas, 28 µg/ml RNase A and 70 U/ml RNase T1) for 15 min at 37 °C prior to antibody incubation. RNA digestion was confirmed on a 1% agarose gel by electrophoresis.

### Quantification of co-immunoprecipitation

Densitometry-based analyses were employed. For each protein in an experiment, an area encompassing the maximum band size was selected and mean intensity of this area was measured using the ‘Histogram’ function in Photoshop. Mean intensities of interacting proteins from control IgG immunoprecipitates were subtracted from those of respective test immunoprecipitates followed by normalization to the mean intensity of the target of the immunoprecipitating antibody.

### Whole cell extracts and western blotting

Cells were washed in cold PBS and lysed in 5 mM Tris pH 7.4, 75 mM NaCl, 0.5 mM EDTA, 0.5% Triton-X-100 with protease inhibitors. Lysate was then spun down at 1000× *g*, 5 min at 4 °C to estimate the protein content of supernatants. Where noted, the cells were lysed in RIPA lysis buffer (10 mM Tris pH 7.4, 100 mM NaCl, 1 mM EDTA, 1% NP-40, 0.5% NaDOC, 0.1% SDS, 10 µg/ml PMSF with protease inhibitors) and spun down at 15,000× *g*, 10 min at 4 °C. Mouse cerebellar and hippocampal tissues in NP40 lysis buffer were vortexed vigorously with two stainless steel beads per tissue (SSB32, Next Advance) every 10 min for 1 h, followed by end-to-end toppling at 4 °C for 1 h. Homogenates in NP40 lysis buffer were then centrifuged at 6000× *g*, 10 min at 4 °C. For western blotting, equal amounts of protein samples were resolved on 10% (w/v) acrylamide gels, transferred to PDVF membrane (IPVH00010, EMD Millipore), blocked with 5% milk in TBST (10 mM Tris-HCl pH 8, 150 mM NaCl, 0.05% Tween 20) for 1 h and probed with primary antibody in TBST overnight at 4 °C. All antibodies were used at 1/1000 dilution. Blots were then washed in TBST, probed with HRP labeled secondary antibodies (goat anti-mouse, goat anti-rabbit and donkey anti-goat), washed again in TBST and imaged with HRP substrate (WBLUR0100A, EMD Millipore) on an ImageQuant LAS 4000 system (GE Healthcare). Mean intensity levels were measured for densitometry-based normalization to tubulin levels. For each protein in an experiment, an area encompassing the maximum band size was selected and mean intensity of this area was measured using the Photshop ‘Histogram’ function. Full size uncropped blots are shown in Supplementary Fig. 7. Antibodies used in the study are listed in Supplementary Table 12.

### p62 and SMN protein purification

P62 and SMN1 expressing plasmids were ordered from GenScript with the codon optimized for overexpression in E. coli. The DNA constructs contain a BamH1 and a Xho1 restriction site flanking each gene. After amplification of the vectors, the plasmids were digested with BamH1 and Xho1 and the DNA fragments were sub-cloned in a vector enabling the expression of P62 and SMN1 as TEV cleavable GST fusion proteins^58^. pGST2-p62 and pGST2-SMN1 were transformed in Rosetta cell and plated on amp-chloramphenicol. Pre-cultures were set up from colonies to grow overnight in 50 ml LB at 37 °C. Culture volumes were increased to 500 ml and grown until OD 0.5. Temperature was then reduced to 18 °C for induction with 0.1 mM IPTG (800–050-IG, Wisent) overnight. 1 L cultures were spun down; pellets were resuspended in binding buffer (50 mM NaPi pH 7, 500 mM NaCl, 10% Glycerol), sonicated thrice 1 min each at power 7 and centrifuged at 27,216× *g* for 30 min, 4 °C. Soluble fractions were incubated with GST beads for 90 min at 4 °C. For p62, beads were then washed and eluted in 10 mM glutathione, 50 mM Tris pH 8.6, 150 mM NaCl for 1 h at 4 °C. The eluate was dialyzed overnight with and without TEV in 50 mM Tris pH 8, 150 mM NaCl, 0.5 mM EDTA, and 5 mM β-mercaptoethanol. Uncleaved GST-p62 (0.81 μg/μl) was frozen in 10% glycerol. Cleaved p62 was separated from GST by gel filtration and frozen in 10% glycerol (0.21 μg/μl). For SMN1, the beads were washed and eluted overnight in 1 M NaCl, 50 mM Tris pH 7 at 4 °C to yield recombinant SMN1 (0.9 μg/μl). The protein was frozen in 10% glycerol.

### GST-pulldown assay with recombinant proteins

Recombinant p62 was mixed in buffer A (20 mM HEPES pH 7.9, 150 mM NaCl, 0.5 mM EDTA, 1 mM DTT, 10% glycerol, 0.1% triton-X-100) and pre-cleared with 5 μl of a 50% slurry of glutathione-agarose beads(16100, Pierce) and 5 μg of GST for 2 h at 4 °C with end-over-end mixing. Following centrifugation at 9000× *g*, 10 min, 4 °C, pre-cleared supernatants were collected and incubated with 5 μl of a 50% slurry of glutathione-agarose beads and either 0.25 μg of GST or GST-C9ORF72 (H00203228-P01, Abnova) for 1 h at 4 °C. Upon precipitation, the mixtures were washed five times with buffer A, 2 min spins at 900× *g*.

Recombinant GST or GST-p62 was bound to glutathione-agarose beads in buffer B (50 mM Tris pH 7.5, 200 mM NaCl, 0.1% triton-X-100) by end-to-end toppling at 4 °C for 1 h 30 min. Beads were previously blocked with 3% BSA in PBS (overnight at 4 °C). Following the incubation, bound beads were spun down at 9000× *g*, 5 min, 4 °C, and washed three times for 5 min each with buffer B, 1 min spins at 9000× *g*. Recombinant SMN was pre-cleared with blocked glutathione-agarose beads bound to GST for 20 min at 4 °C. Following centrifugation at 9000× *g*, 5 min, 4 °C, pre-cleared supernatant was collected and incubated with previously prepared glutathione-agarose beads bound to GST or GST-p62 for 1 h at 4 °C. Upon precipitation, the mixtures were washed five times with buffer B, 1 min spins at 9000× *g*.

The bound proteins were then analyzed by SDS-PAGE.

### Peptide-pulldown assay

The peptide—KGRGRGRGRG was custom ordered from GenScript in two forms: all 4 arginines (i) symmetrically dimethylated, or (ii) asymmetrically dimethylated. A volume of 1 mg of each of the peptides in 400 μl of PBS was incubated with 400 μl of Affi-Gel 10 (1536099, Bio-Rad) media washed in cold deionized water to make 50% slurry. The uniform suspension was left to rotate on a wheel overnight at 4 °C. A volume of 40 μl of 1 M ethanolamine HCl in 0.5 M Tris pH 8 was added to the slurry to block any active esters and left rotating for 2+ h at 4 °C. Peptide bound media was then washed in 500 μl PBS thrice, resuspended in 1 ml of 0.1% sodium azide in PBS, and stored at 4 °C. For the pulldown assays, affi-gel 10 media—10 μl per reaction was washed in PBS thrice and resuspended in 1 ml of binding buffer (20 mM HEPES pH 7.9, 200 mM NaCl, 10% Glycerol, 0.05% Tween 20) prior to pre-clearing 375 ng of recombinant p62 and/or SMN for 20 min at 4 °C. Pre-cleared proteins were separated from the beads upon centrifugation at 9000× *g*, 5 min. As a positive control, 1% of the supernatant was frozen; remaining was incubated with 30 μl of affi-gel 10 media bound to (i) no peptide, (ii) symmetrically dimethylated, or (iii) asymmetrically dimethylated RG(x4) peptide, on a rotating wheel for 1 h at 4 °C. Mixture was then washed six times, each in 500 μl of binding buffer with 5 min rotations at 4 °C and 9000× *g*, 1 min spins at 4 °C. SDS-sample buffer was added and the reactions were boiled at 99 °C for 5 min prior to western blot analysis.

### In vitro methylation assay

- **Substrate and enzyme preparation:** GST-FUS-WT (44978, Addgene) and pGST2 with no insert were transformed in BL21 strain. Colonies were picked to grow overnight cultures in 50 ml LB at 37 °C. Culture volumes were increased to 1000 ml and grown at 37 °C until OD 0.5. Protein expression was induced with 0.1 mM IPTG for 5 h at 37 °C. Cultures were spun down at 1700× *g* for 20 min, pellets lysed in PBS containing protease inhibitors, sonicated five times 15 s each with 10 s intervals at power 3, and centrifuged at 15,309× *g* for 20 min at 4 °C. Supernatants were solubilized in 1% Triton-X-100 for 30 min, 4 °C and incubated with 500 μl GST beads (16100, Pierce) overnight at 4 °C. Washed beads were then stored in PBS at 4 °C. Active human PRMT5 in complex with MEP50 was purchased (SRP0145, Sigma-Aldrich).
- **Enzymatic reactions:** Equal amounts of protein on agarose beads were each incubated with 1 μg PRMT5-MEP50 and SAM in a 30 μl reaction buffer (50 mM Tris pH 8, 1 mM DTT, 10 mM NaCl) for 1 h at 30 °C and 3 h at 37 °C. For western blot and mass spectrometry analyses, 80 uM cold SAM (A7007, Sigma-Aldrich) was used while 1 µCi SAM[3 H] (NET155V250UC, PerkinElmer) was used per radioactive reaction. Reactions were stopped by adding SDS-sample buffer. To detect radioactive signal, samples transferred to PVDF membrane were dried 1 h, sprayed thrice with EN^3^HANCE Spray (6NE970C, PerkinEmer), while allowing drying for 30 min and rotating 90° following each spray. Tritium signal was further enhanced with Transcreen LE (Z374253, Sigma-Aldrich) during the 2-week exposure at −80 °C. Alternatively, radioactive polyacrylamide gels were soaked in EN^3^HANCE liquid (6NE9701, PerkinElmer) for 30 min at room temperature, washed six times in water (5 min × 3 times and 15 min × 3 times), vacuum dried for 3 h at 60 °C and exposed for 2 weeks at −80 °C.

### Nuclear/cytoplasmic subcellular fractionation

Cells washed with cold PBS were lysed in hypotonic lysis buffer (10 mM HEPES pH 7.9, 10 mM KCl, 1.5 mM MgCl_2_, 0.5 mM DTT, protease inhibitors), incubated on ice for 10 min and homogenized with 15 strokes of a Dounce glass homogenizer. Supernatant was collected as the cytoplasmic extract after centrifuging the lysate at 2000× *g* for 10 min. To collect the nuclear extract, the pellet was washed in hypotonic lysis buffer, further centrifuged at 2000× *g* for 10 min, resuspended in NP-40 buffer (50 mM Tris pH 8, 400 mM NaCl, 5 mM EDTA pH 8, 1% NP-40, 0.2% SDS) and sonicated for 15 s at power 3. For western blotting, the fractions were run in equal volume proportions. For quantification, cytoplasmic fractions were normalized to GAPDH while nuclear fractions were normalized to methylated histone H3 on lysine 27—H3K27.

### Quantification of nuclear/cytoplasmic fluorescence

DAPI nuclear staining and brightfield images were used respectively to draw nuclear and cytoplasmic regions of interest on individual cells. Mean fluorescence intensity was measured for each of the two regions per cell using Fiji and thereby represented as a ratio.

### Mass spectrometry

Chemicals used include urea, dithiothreitol (DTT), ammonium bicarbonate (ABC), and iodoacetamide (IAA), all of which were purchased from Sigma-Aldrich (St. Louis, MO). HPLC grade water and acetonitrile (ACN) were from J.T Baker. Formic acid (FA) and citric acid were obtained from Merck (Darmstadt, Germany). Trypsin was purchased from Promega (Madison, WI). All the chemicals were of analytical purity grade except ACN and FA, which were of HPLC grade.

Sliced protein bands were digested as reported previously^59^. Briefly, gel slices were cut into smaller pieces, shrunk using 100% ACN, then reduced and alkylated sequentially. Trypsin solution of 10 ng/µL was used to cover the gel pieces for digestion overnight. Tryptic peptides were then extracted using 80% ACN. The solution was then dried down and reconstituted in 20 µL 0.5% formic acid, and 4 µL was loaded for MS analysis.

LCMS Analysis: Eksigent 2D + nanoLC system (Dublin, CA) was connected to a Q-Exactive mass spectrometer (Thermo Electron, Waltham, MA), equipped with a nano-electrospray interface operated in positive ion mode. The solvent system consists of buffer A of 0.1% FA in water, and buffer B of 0.1%FA in 80% acetonitrile. Dried down protein digests were acidified with 0.5% (v/v) formic acid and loaded on a 75 μm I.D. × 150 mm fused silica analytical column packed in-house with 1.9 μm ReproSil-Pur C18 beads (100 Å; Dr. Maisch GmbH, Ammerbuch, Germany) at a flow rate of 500 nL/min for 15 min. Then the flow rate was changed to 200 nL/min to perform the peptide separation. Gradient elution was set as 5–35% buffer B per hour. The spray voltage was set to 2.0 kV and the temperature of the heated capillary was set to 300 °C. The instrument method consisted of one full MS scan from 300 to 1800 m/z followed by data-dependent MS/MS scan of the 12 most intense ions, a dynamic exclusion repeat count of 1 in 30 s, and an exclusion duration of 30 s. The full mass was scanned in an Orbitrap analyzer with *R* = 70,000 (defined at *m/z* 400) for MS1 and 17,500 for MS2. To improve the mass accuracy, all the measurements in the orbitrap mass analyzer were performed with a real time internal calibration by the lock mass of background ion 445.120025. The charge state rejection function was enabled, and charge states with unknown and single charge state were excluded for subsequent MS/MS analysis. All data were recorded with Xcalibur software (Thermo Fisher Scientific, San Jose, CA).

Data Analysis: The peak lists of the raw files were processed and analyzed with MaxQuant (Version 1.5.2.8)^60^ against UniProt human protein database, including commonly observed contaminants. Cysteine carbamidomethylation was selected as a fixed modification; methionine oxidation, protein N-terminal acetylation and arginine methylation were set as variable modifications. Enzyme specificity was set to trypsin, not allowing for cleavage N-terminal to proline. Up to two missing cleavages of trypsin were allowed. The precursor ion mass tolerances were 7 ppm, and fragment ion mass tolerance was 20 ppm. Razor and unique peptides were used for LFQ quantitation. FDR was set at 0.01 on protein, peptide and modification of specific sites; a minimum length of seven amino acids was used for peptide identification. For protein identification, if the identified peptide sequence of one protein was equal to or contained another protein’s peptide set, these two proteins were grouped together by MaxQuant and reported as one protein group.

### Bioinformatic analyses of splicing and FUS-motif enrichment

To analyze potential splicing events using published microarray data from p62 knockout mice the following MoGene-1_0-st-v1 chip files were used: (GSM1522557, GSM1522558 p62 knockout. GSM1522560 GSM1522562 wild type). The method outlined in^61^ with Affymetrix Power Tools 1.17.0 and MoGene-1_0-st-v1.r4 annotation files was used to identify splicing candidate genes. Genomic locations for probesets were taken from Affymetrix annotations: MoGene-1_0-st-v1.na35.mm10.probeset.csv. Genomic locations for exons and genes, as well as gene to probeset associations were downloaded from ENSEMBL BioMart mmusculus_gene_ensembl, dec2015.archive.ensembl.org. Genomic introns sequences were determined by selecting the genomic interval of each ENSEMBL gene with status “known” and removing intervals for exons associated with that gene. Genes were considered putatively spliced if they had MIDAS *p*-values < 0.01. Instances of the motif GTGGT identified as the most frequent FUS-binding motif^41^ in intronic sequences were then counted and normalized to the total sequence length of mRNAs.

The list of 957 mRNAs whose splicing was putatively affected by FUS was taken from Table 6 in^41^ and 862 were found to be potentially mappable to the above derived genes based on MGI gene symbols in the ENSEMBL tcid annotations. 1481 putatively spliced MGI genes were identified in our analysis; there are 22,509 unique MGI genes in the microarray gene expression set. Ninety-seven of the genes whose splicing was putatively affected by FUS^41^ were also found among the 1481 putatively spliced genes in p62 knockout mice. The predicted probability distribution was generated using the R dhyper function and the above parameters. The probability of seeing 97 or greater overlapping gene symbols by chance is 1.57e-07. The cumulative fraction of -log10 of the best probeset MIDAS p-value per gene was plotted for all genes, as well as for the gene set matching FUS targets and those with MIDAS *p*-values 0.01. Additionally, the cumulative density of -log10 of the best probeset MIDAS p-value per gene was plotted for all genes, as well as the set with higher FUS motif (GTGGT^41^) density than the mean (*p*-value 0.01) by a one-sided proportion test.

### Analysis of putative interactomes of C9ORF72 and P62

Protein intensity values obtained from MaxQuant were used for the statistical confidence assessment of C9ORF72 and P62 interactions by SAINTq^62^. For each protein, SAINTq compares its intensity values in the biological replicates of the immunoprecipitations (IPs) against those in the IgG control replicates under the same experimental conditions. SAINTq then assigns each protein a Bayesian False Discovery Rate (BFDR) that is based on the level of significance of the intensity difference between the immunoprecipitations and the controls. Proteins with a BFDR < 0.05 were considered as high-confidence protein-protein interactions. C9ORF72 and P62 interacting proteins were intersected with known stress granule proteins^22^ and ALS-linked genes^63^. Interactomes were also overlapped with published datasets of arginine dimethylated proteins and potential PRMT5 substrates^44,64–67^. Potential PRMT5 substrates from Supplementary Table 1 of^65^ which had SILAC H/L ratio > 1.02 for at least two out of the three following treatment conditions: (i) EPZ015666—selective PRMT5 inhibitor, (ii) siPRMT5_Exp1, and (iii) siPRMT5_Exp2 were included. Cytoscape 3.4.0^68^ plugin BiNGO was used to perform GO term analyses. Overrepresented categories were chosen after Benjamini & Hochberg False Discovery Rate correction. Hypergeometric statistical test was used to ascertain the *p*-value of GO term enrichment.

### Silver staining

Immunoprecipitations of P62, FUS, and C9ORF72 were performed as described above except in a sterile environment to reduce keratin contamination. Following the final washes in NP-40 lysis buffer, magnetic beads were resuspended in 1% SDS (in lysis buffer), boiled at 99 °C for 5 min; then DTT was added to 10 mM and samples were boiled at 99 °C for 5 min prior to silver staining. Samples were run on precast protein gels (4561094, Bio-Rad) and processed for silver staining as per manufacturer’s instructions (17-1150-01, GE Healthcare). Protein lanes were then excised for trypsin digestion.

### MTT Assay

Vybrant™ MTT Cell Proliferation Assay Kit (V13154, Thermo Fisher Scientific) was employed in accordance with manufacturer’s guidelines.

### Patient information, tissue collection, and consent

Patient tissues were collected with informed consent with approval from the Sunnybrook Health Sciences Centre Research Ethics Board and the Ottawa Hospital Research Ethics Board. Specimens were also provided by the Department of Veterans Affairs Biorepository. Overall, two non-ALS control cases, five ALS cases carrying C9ORF72 repeat expansions, and three ALS cases not carrying C9ORF72 repeat expansions were used in the study.

### Statistical analysis

Statistical analyses were performed using two-tailed Student’s *t*-tests in Microsoft Excel. A *p*-value of <0.05 was considered statistically significant. Significance was denoted as follows: *p-value < 0.05, **p-value < 0.01, ***p-value < 0.001.

### Data availability

The mass spectrometry proteomics data have been deposited to the ProteomeXchange Consortium via the PRIDE partner repository with the dataset identifiers PXD009759, PXD009741 and PXD009760. Dataset legends and reviewer account details are included in the Supplementary Information. The authors declare that all data supporting the findings of this study are available within the article and its Supplementary Information files, or from the corresponding author upon reasonable request.

## Electronic supplementary material

Supplementary Information

Description of Additional Supplementary Files

Supplementary Data 1

Supplementary Data 2

Supplementary Data 3
