# Supplementary material for: A complex of C9ORF72 and p62 uses arginine methylation to eliminate stress granules by autophagy
Source: Nat Commun. 2018 Jul 18;9:2794. doi: 10.1038/s41467-018-05273-7 (PMC6052026; doi:10.1038/s41467-018-05273-7)
Supplement: Supplementary file 1 — Supplementary Information [file 41467_2018_5273_MOESM1_ESM.pdf]

Supplementary Information

A complex of C9ORF72 and p62 uses arginine methylation to eliminate stress granules by autophagy

Chitiprolu et al.

Supplementary Figures

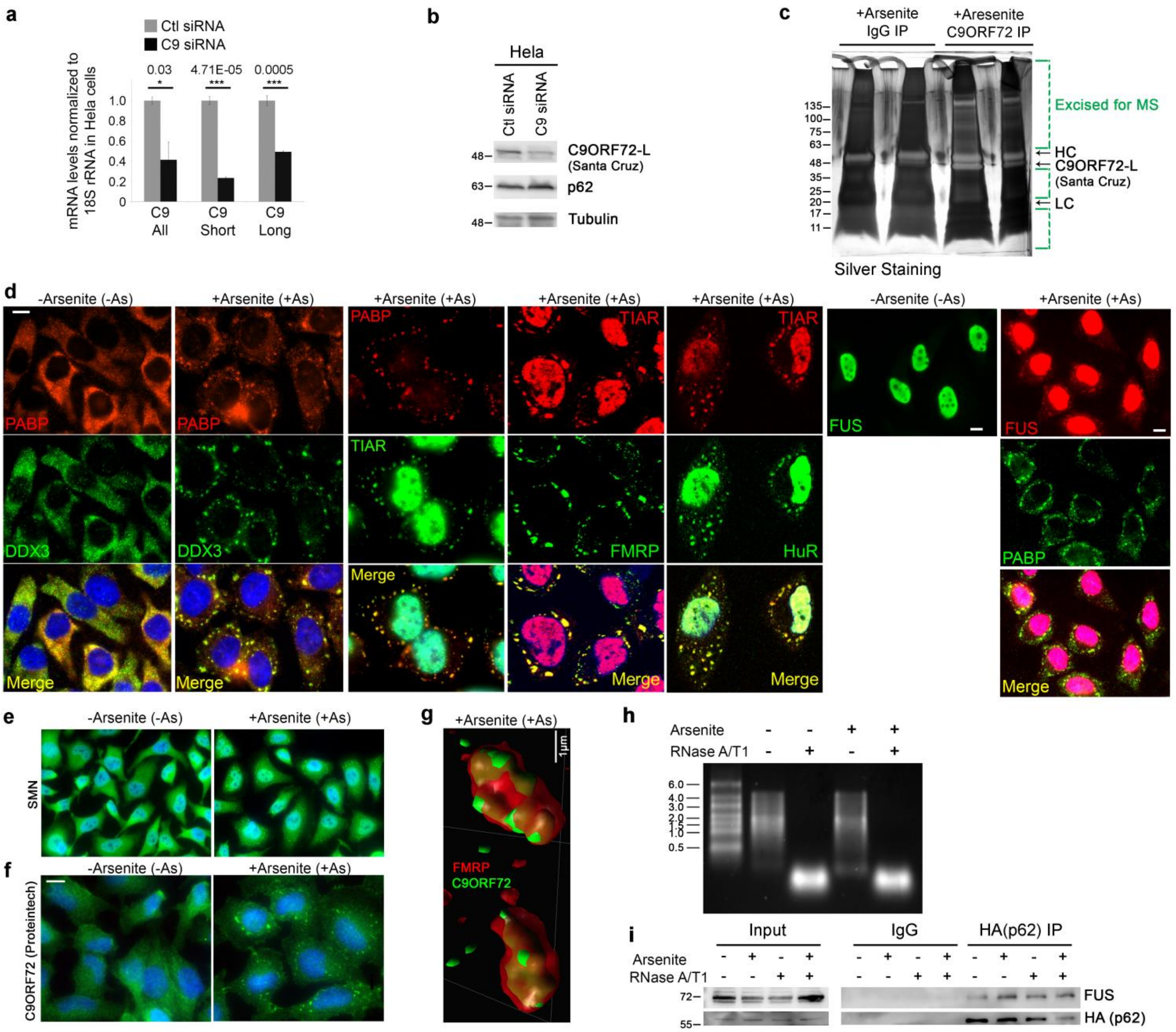

Supplementary Figure 1

Supplementary Figure 1. C9ORF72 localizes to stress granules

(a) RT-qPCR of C9ORF72 in cells treated with siRNA targeting control (Ctl) or *C9ORF72* (C9); RT-qPCR primers were designed to detect all *C9ORF72* mRNA, or only the long (C9 long) or short isoforms (C9 short) of *C9ORF72* mRNA.

(b) Western blot of C9ORF72 in cells treated with control or C9ORF72 targeting siRNA; C9ORF72-L indicates C9ORF72 long isoform.

(c) Silver stained gel of C9ORF72 immunoprecipitates from lysates treated with arsenite (As) prior to mass spectrometry; two samples of IgG control immunoprecipitations and C9ORF72 immunoprecipitations are included on the representative gel; regions of gel excised for mass spectrometric analyses are highlighted with green dashed lines.

(d) Immunofluorescence of stress granules upon oxidative stress using various markers (PABP, TIAR, FMRP, DDX3, HuR, FUS) to demonstrate the specificity of labeling with the antibodies and the ability to use alternate stress granule markers.

(e) Immunofluorescence of SMN in cells untreated or treated with arsenite.

(f) Immunofluorescence of C9ORF72 in cells untreated or treated with arsenite.

(g) 3-D reconstruction of Z-stacks acquired by confocal microscopy of cells treated with arsenite and labeled with C9ORF72 and FMRP.

(h) Ethidium bromide stained agarose gel showing total cellular RNA subjected to identical RNAse treatment as used to treat immunoprecipitates in (i).

(i) Western blot of HA-p62 immunoprecipitates from lysates with and without arsenite treatment as indicated (+, -); immunoprecipitates were treated with a combination of RNAse A and T1 or treated with vehicle control as indicated.

HeLa cells were used in all experiments. Scale bar = 10 µm.

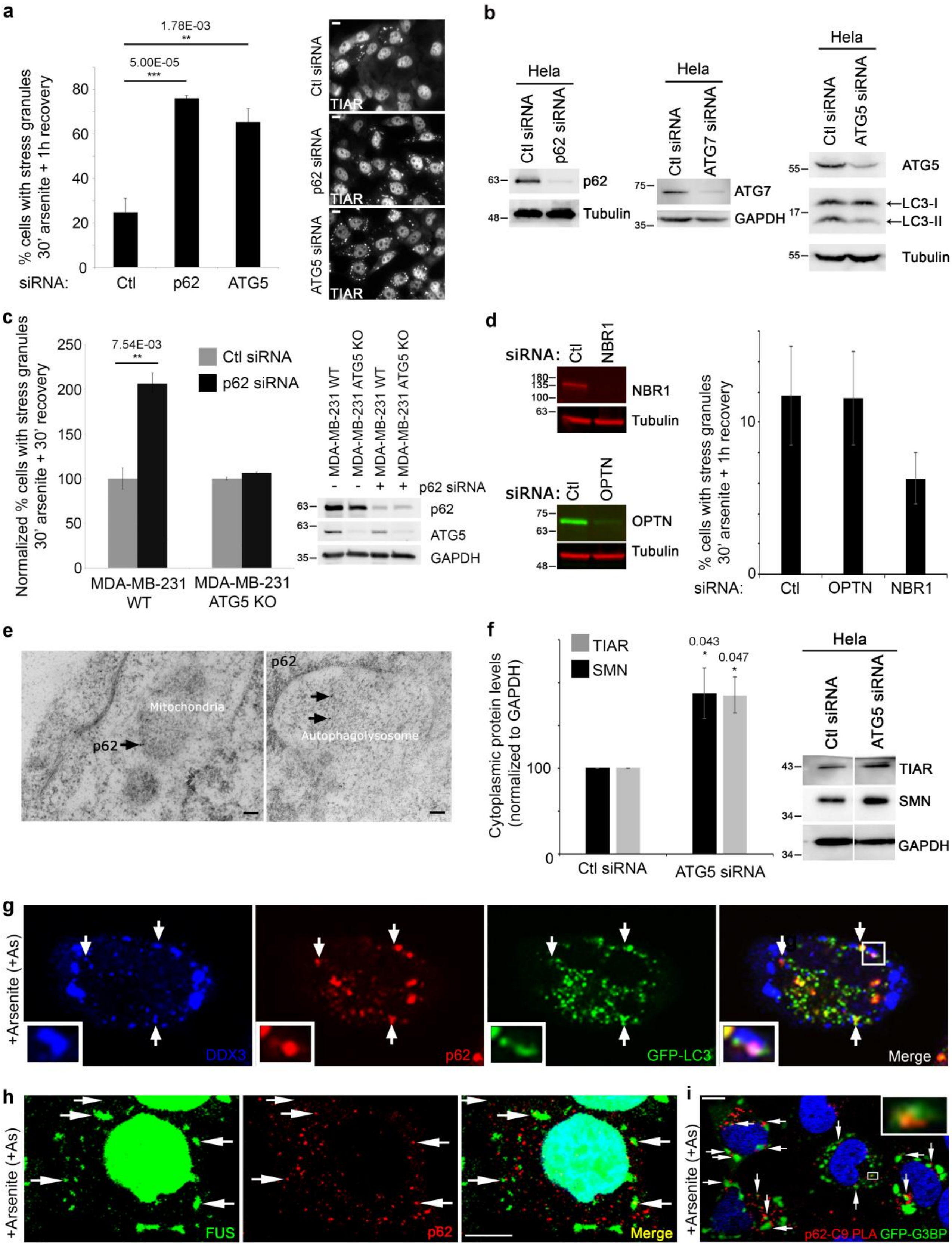

Supplementary Figure 2

Supplementary Figure 2. P62 docks on stress granules and is required for their elimination by autophagy

(a) Percentage of cells containing stress granules following 1 h of recovery from 30 minutes treatment with arsenite upon transfection with siRNA targeting p62, ATG5 or control siRNA (Ctl); Right, representative images.

(b) Western blot of p62, ATG7 and ATG5 in cells treated with control siRNA (Ctl) or siRNA targeting p62, ATG7 or ATG5.

(c) Percentage of wild-type or ATG5 knockout MDA-231 cells containing stress granules following 30 minutes of recovery from 30 minutes aresnite treatment upon transfection with siRNA targeting p62 or control siRNA (Ctl); Right, western blot validating knockdown of p62 and knockout of ATG5 in MDA-231 cells.

(d) Left, western blot validating knockdown of NBR1 (top) and OPTN (bottom); Right, percentage of cells containing stress granules in cells treated with control (Ctl), OPTN or NBR1 siRNA after one hour recovery from 30 minutes arsenite (n=3, mean ± SD, Student's *t*-test).

(e) Immuno-electron micrographs of p62; mitochondria (mito) and autophagolysosomes are indicated. Scale bar = 100 nm.

(f) Left, quantification of TIAR and SMN cytoplasmic levels in cells treated with siRNA targeting ATG5 or control siRNA (n=5, mean ± SD, Student's *t*-test); Right, representative western blot.

(g) Immunofluorescent microscopy of arsenite treated cells labeled with antibodies recognizing DDX3, endogenous p62 and expressing GFP-LC3.

(h) Immunofluorescent microscopy of arsenite treated cells labeled with antibodies recognizing endogenous FUS and p62.

(i) Fluorescent microscopy image of proximity ligation assay for association of C9ORF72 and p62 in cells expressing the stress granule marker GFP-G3BP.

HeLa cells were used in all experiments. Scale bar = 10 µm.

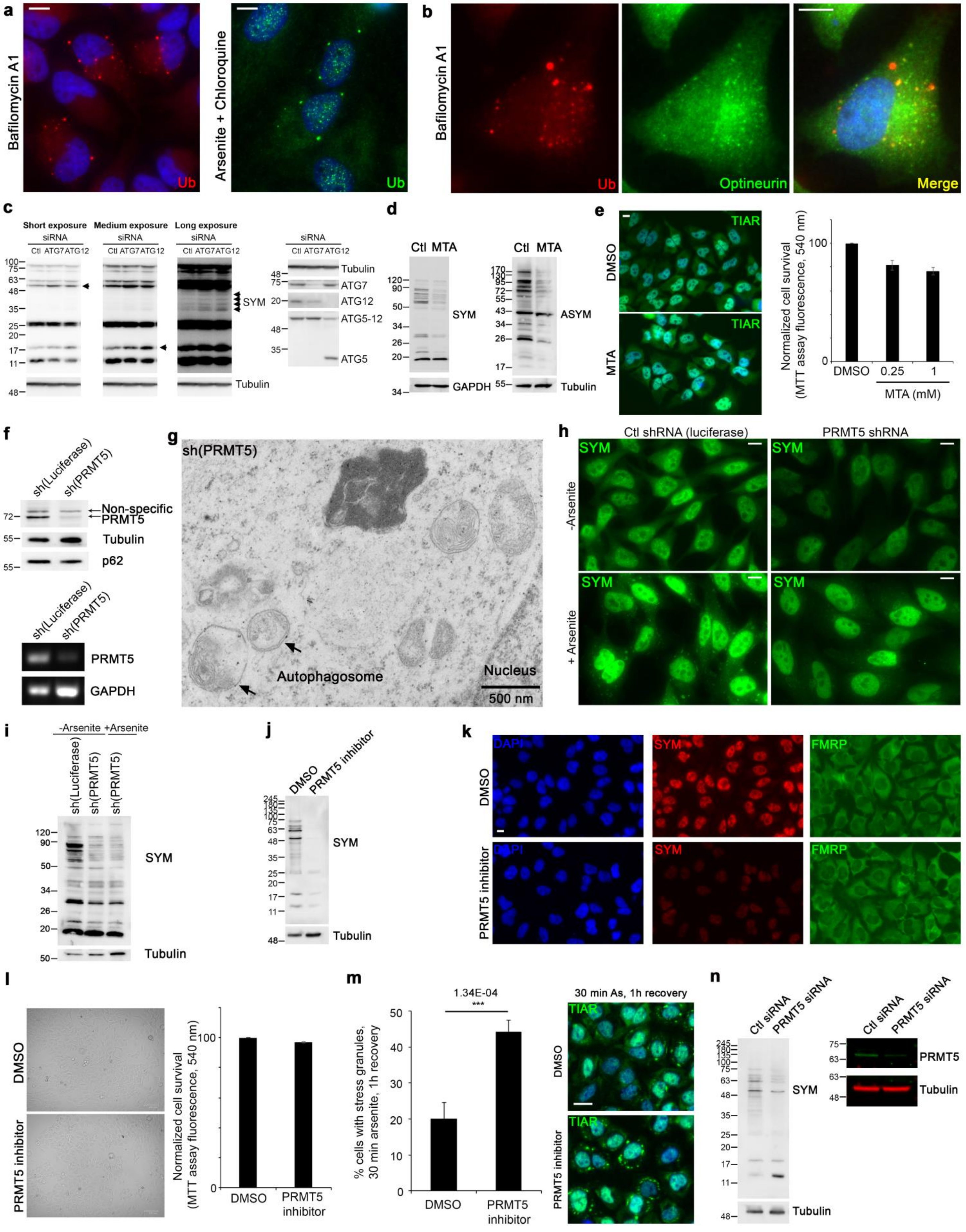

Supplementary Figure 3

Supplementary Figure 3. Stress granules are enriched in proteins modified with symmetrically dimethylated arginines

(a, b) Immunofluorescent microscopy of cells treated with (a) Bafilomycin A1 (left) or Arsenite and chloroquine (right) and labeled with Ubiquitin antibody and (b) Bafilomycin A1 and co-labeled with Ubiquitin and Optineurin.

(c)) Left, western blot of proteins containing symmetrically (SYM) dimethylated arginines in total lysates of HEK293T cells treated with siRNA targeting *ATG7, ATG12*; Right, western blot confirming ATG7, ATG12 knockdown.

(d) Western blot of proteins containing symmetrically (SYM) and asymmetrically (ASYM) dimethylated arginines in total lysates of cells treated with vehicle or methylthioadenosine (MTA).

(e) Left, immunofluorescent microscopy showing lack of stress granule induction in cells treated with vehicle or methylthioadenosine (MTA); Right, quantification of cell viability by MTT assay in cells treated with vehicle or methylthioadenosine.

(f) Left, western blot of PRMT5 and p62 in cells stably transduced with lentiviruses packaging shRNA targeting *PRMT5* (shPRMT5) or control shRNA targeting luciferase (shLuciferase); Right, RT-PCR of *PRMT5* mRNA in cells stably transduced as above.

(g) Electron microscopy of autophagosomes (indicated by arrows) in shPRMT5 cells.

(h) Immunofluorescent microscopy of proteins containing symmetrically dimethylated arginines (SYM) in shPRMT5 or shLuciferase cells treated or not with arsenite.

(i) Western blot of total lysates from shPRMT5 or shLuciferase cells treated or not with arsenite and labelled with antibody specific for symmetrically dimethylated arginines.

(j) Western blot of proteins containing symmetrically (SYM) dimethylated arginines in total lysates of cells treated with vehicle or PRMT5 inhibitor (EPZ015666).

(k) Immunofluorescent microscopy showing lack of stress granule (FMRP) induction and diminished symmetric dimethylation signals in cells treated with vehicle or PRMT5 inhibitor.

(l) Left, light microscopy of healthy cells upon treatment with PRMT5 inhibitor; Right, quantification of cell viability by MTT assay in cells treated with PRMT5 inhibitor.

(m) Left, percentage of cells containing stress granules following 1 hour recovery from 30 minutes arsenite treatment in the presence of PRMT5 inhibitor (n=4, mean ± SD, Student's *t*-test); Right, representative images.

(n) Left, western blot of proteins containing symmetrically (SYM) dimethylated arginines in total lysates of cells treated with siRNA targeting *PRMT5*; Right, western blot confirming PRMT5 knockdown.

HeLa cells were used in all experiments. Scale bar = 10 μm.

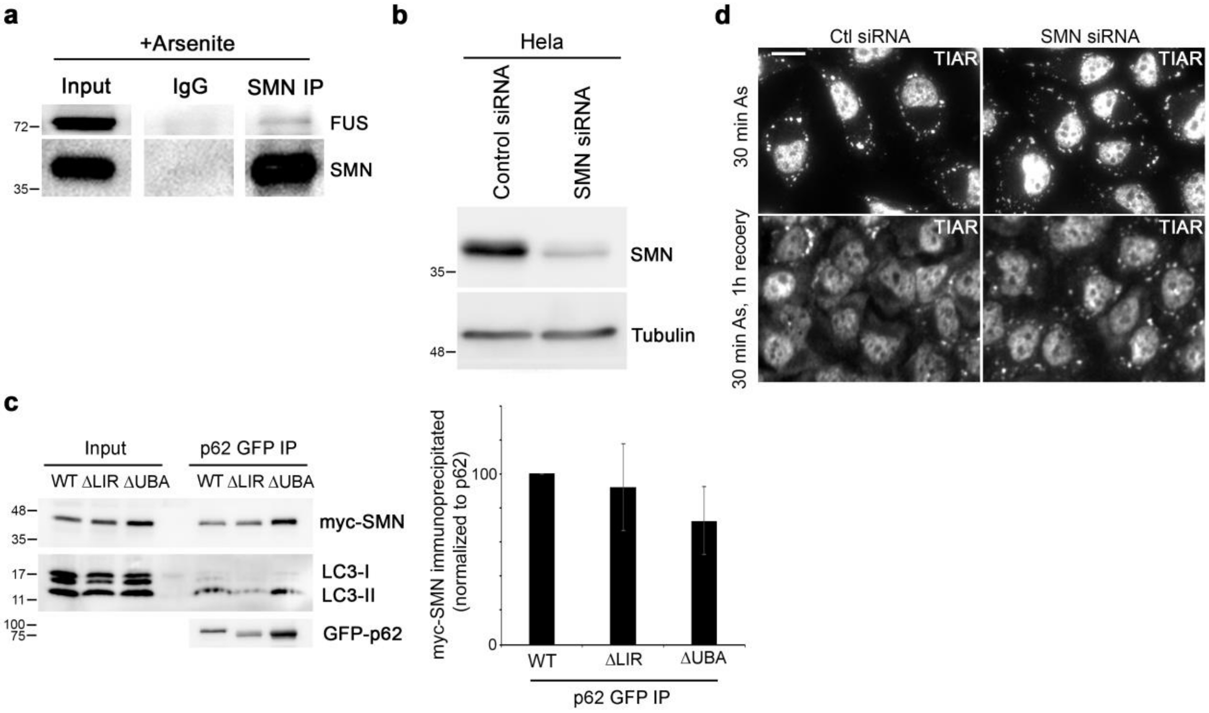

Supplementary Figure 4

Supplementary Figure 4. FUS associates with SMN

(a) Western blot of FUS in immunoprecipitates of SMN from cells treated with arsenite.

(b) Western blot of SMN in cells treated with control siRNA or siRNA targeting *SMN*.

(c) Left, western blot of immunoprecipitates of wild-type (WT) and p62 mutants; Right, quantification of myc-SMN pulled down with indicated p62 expression constructs (n=2).

(d) Representative images of cells transfected with control (Ctl) or SMN siRNA containing stress granules (TIAR) following 30 min arsenite treatment or following 1 hour recovery from arsenite. HeLa cells were used in all experiments. Scale bar = 10 µm.

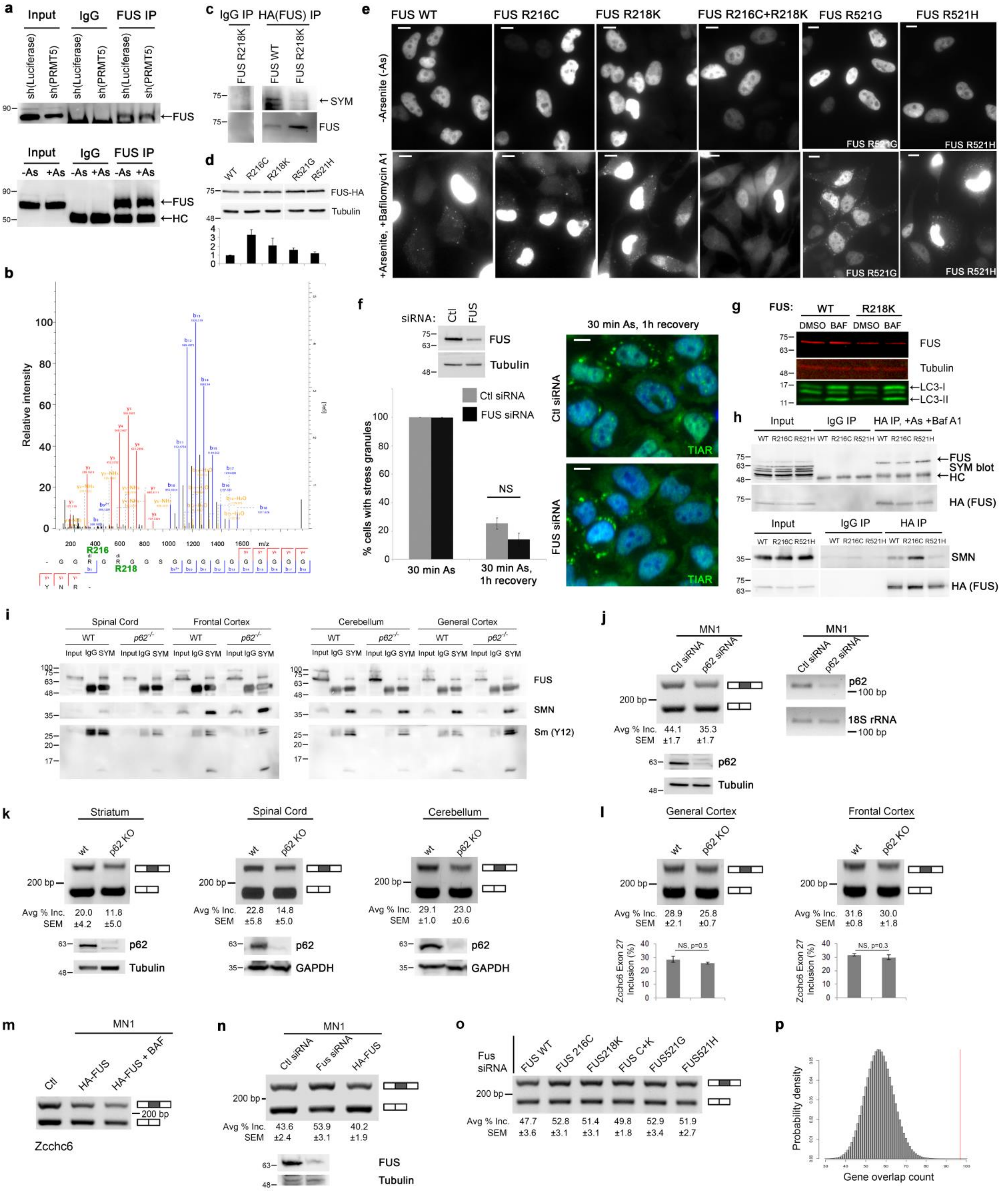

Supplementary Figure 5

Supplementary Figure 5. FUS mutants distribute in the cytoplasm of cells and exert splicing defects shared with p62 depleted cells and mice

(a) Western blot of FUS immunoprecipitates from HeLa cells used for MS/MS; Top, cells expressing shRNA targeting PRMT5 (shPRMT5) or luciferase control (shLuciferase); Bottom, cells treated with arsenite (+As) or vehicle (-As).

(b) Annotated spectrum from LC-MS/MS of dimethylated FUS peptide 214-GGRGRGGSGGGGGGGGGYNR-234.

(c) Western blot for symmetrically dimethylated arginines (SYM) in immunoprecipitates of wild-type or R218K HA-FUS HeLa cells (n=3-6, mean ± SD).

(d) Western blot of wild-type and mutant FUS in HeLa cells.

(e) Immunofluorescent microsocopy of wild type or FUS mutants in HeLa cells.

(f) Top, western blot of FUS in HeLa cells treated with FUS targeting siRNA; Left, percentage of transfected cells containing SGs following 30 min arsenite or 1 hour recovery from arsenite (n=2, mean ± SD, Student's *t*-test); Right, representative images.

(g) Western blot of FUS wild-type and R218K mutant upon Bafilomycin A1 treatment of HeLa cells.

(h) Western blot of immunoprecipitates of wild-type and FUS mutants from HeLa cells treated with arsenite and Bafilomycin A1.

(i) Western blot of immunoprecipitates of symmetrically dimethylated proteins from wild-type and *p62*$^{-/-}$ mice; Sm (Y12) serves as a positive control.

(j) Left, RT-PCR of splice variants of Zcch6 mRNA in MN1 cells treated with siRNA; Right, RT-PCR and Bottom, western blot of p62 in the corresponding cells.

(k) Top, RT-PCR of splice variants of Zcch6 mRNA from wild-type or *p62*$^{-/-}$ mice; Bottom, western blot of p62.

(l) Top, RT-PCR of splice variants of Zcch6 mRNA from wild-type or *p62*$^{-/-}$ mice; Bottom, quantification from 3 mice.

(m) RT-PCR of splice variants of Zcch6 mRNA in MN1 cells mock transfected, transfected with HA-FUS and/or treated with Bafilomycin A1.

(n) Top, RT-PCR of splice variants of Zcch6 mRNA in MN1 cells treated with control siRNA or siRNA targeting FUS or transfected with HA-FUS; Bottom, western blot of FUS.

(o) RT-PCR of splice variants of Zcch6 mRNA in MN1 cells treated with siRNA targeting FUS 3'UTR and co-transfected with wild-type or FUS mutants.

(p) Normal probability distribution of number of mRNAs bound to FUS and alternatively spliced in *FUS*$^{-/-}$ brain; red line indicates probability of observed overlap with mRNAs with putative alternative splicing in *p62*$^{-/-}$ brain. Scale bar = 10 µm.

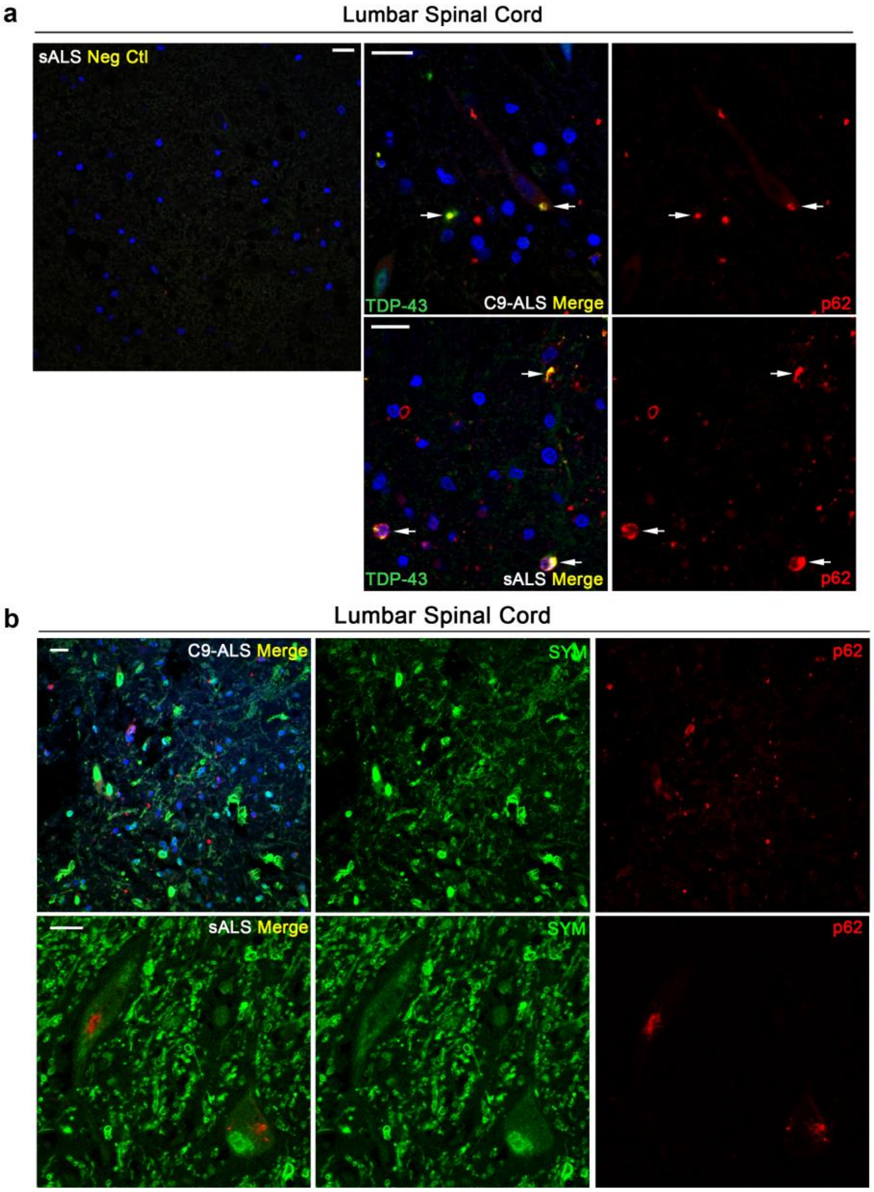

Supplementary Figure S6

Supplementary Figure 6. Lumbar spinal cord of ALS patients with sporadic disease or C9ORF72 repeat expansions exhibit p62+ inclusions that are not enriched in symmetrically dimethylated arginines

(a) Two-color immunofluorescence staining of lumbar spinal cord sections from patients with C9ORF72 repeat expansions (C9-ALS) or sporadic ALS (sALS) with isotype control non-specific antibody or antibodies to p62 and TDP-43 (Left, merged image); arrows represent inclusions and co-localization.

(b) Two-color immunofluorescence staining of lumbar spinal cord sections of patients with C9ORF72 repeat expansions (C9ORF72) and sporadic ALS (sALS) with antibodies to p62 and proteins with symmetrically dimethylated arginines (SYM). Scale bar = 20 μm.

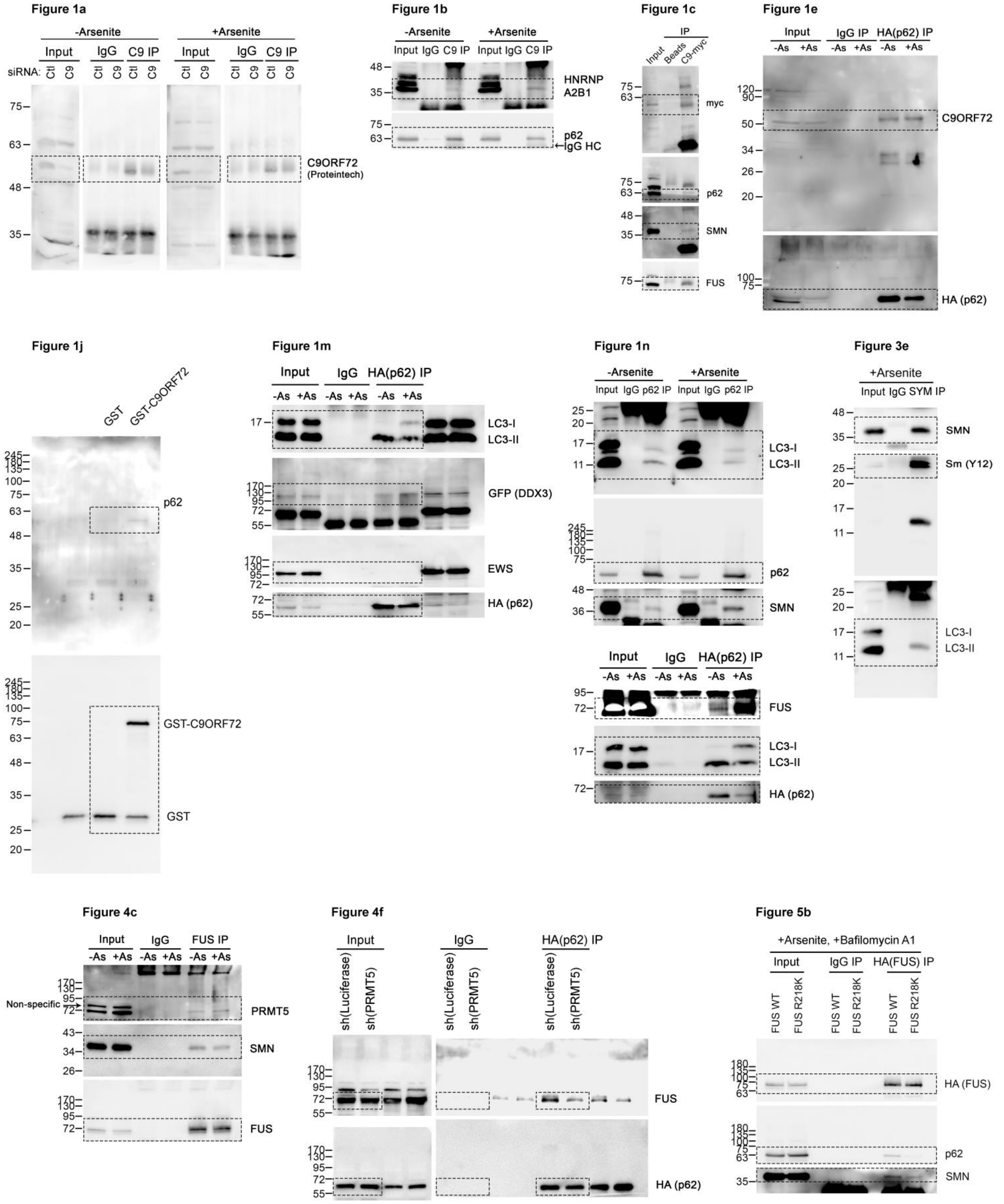

Supplementary Figure 7

Supplementary Figure 7. Uncropped western blot images

Uncropped blots are shown for key experiments including co-immunoprecipitations and GST-pulldown assays. Molecular weight markers for these specific blots are shown only in the uncropped versions (in this figure), unlike all the remaining blots where the size markers are included directly in the figure panels.

Supplementary Tables

Supplementary Tables 1 to 6: Throughout tables below, putative C9ORF72 and p62 interacting proteins were intersected with known stress granule proteins[1] and ALS-linked genes[2]. Interactomes were also overlapped with consolidated published datasets of arginine dimethylated proteins or potential PRMT5 substrates[3-7]. Potential PRMT5 substrates were derived from Supplementary Table 1 of [4] with SILAC H/L ratio > 1.02 for at least two out of the 3 following treatment conditions: (i) EPZ015666 – selective PRMT5 inhibitor, (ii) siPRMT5_Exp1 and (iii) siPRMT5_Exp2.

Supplementary Table 1. C9ORF72 mass spectrometric analyses in arsenite-treated HeLa cells

C9ORF72 Interactors in arsenite-treated HeLa cells						
SAINTq Analysis BFDR < 0.05	Saintq BFDR < 0.05 and enriched in all 3 C9ORF72 IPs	Stress Granule Components[1]	Di-methylated Arginine Proteins	Potential PRMT5 Substrates[4]	Di-methylated R sites interacting with C9ORF72	ALS-linked Proteins[2]
ACTG1;ACTB;ACTA1;ACTG2;ACTA2<br>ACTN1<br>ACTN4<br>ADAR<br>AKR1C2;AKR1C1<br>ALPL<br>ALYREF<br>AP2A1<br>AP2A2<br>AP2B1<br>AP2M1<br>AP2S1<br>ATAD3A;ATAD3B<br>ATP5A1<br>ATXN2L<br>BAG3<br>BANP<br>BCLAF1<br>BST2<br>CACNA2D1<br>CAD<br>CAMK2D<br>CAMK2G;CAMK2B<br>CAPRIN1<br>CAPZA1<br>CASC3<br>CCT2<br>CCT3<br>CCT4<br>CCT7<br>CCT8<br>CD44	ATAD3A;ATAD3B<br>DDX3X;DDX3Y<br>EEF1A1P5;EEF1A1;EEF1A2<br>HIST1H2AC;HIST3H2A;HIST1H2AB<br>HIST1H2BN;HIST1H2BL;HIST1H2BM;HIST1H2BH;HIST2H2BF;HIST1H2BC;HIST1H2BD;H2BFS;HIST1H2BK<br>HIST1H4A<br>HNRNPA1<br>HNRNPA2B1<br>HNRNPC<br>HSPA8<br>ITPR1<br>ITPR3<br>MYH9<br>PABPC1<br>PKM<br>RBMX;RBMXL1<br>RPL10;RPL10L<br>RPL26;KRBA2;RPL26L1<br>RPL27A<br>RPL39P5;RPL39<br>RPS11<br>RPS18<br>RPS4X;RPS4Y2;RPS4Y1<br>SAFB<br>SAFB2	ATAD3A<br>DDX3X<br>HNRNPA1<br>HNRNPA2B1<br>PABPC1<br>SAFB2<br>TUBA1C;TUBA3C<br>TUBB3;TUBB8	EEF1A1<br>HNRNPA1<br>HNRNPA2B1<br>HSPA8<br>PABPC1<br>RBMX;RBMXL1<br>SAFB<br>SAFB2<br>TUBA3C	EEF1A1_R166<br><br>FUS_R218, R259, R394<br><br>HIST1H3A_R64, R129<br><br>HNRNPA1_R206, R218, R232, R225, R194, R213, R196, R215, R265<br><br>HNRNPA2B1_R238<br><br>HNRNPAB_R253, R250, 245, R275, R273<br><br>HNRNPU_R715, R720, R709, R755, R572<br><br>HNRNPUL1_R181, R620<br><br>RBMX;RBMXL1_R144<br><br>TAF15_R373, R293, R206, R286, R185, R338, R308, R315, R331, R195, R301, R124, R348, R137, R234<br><br>TRA2B_R224, R238,	FUS_R216, R218<br><br>G3BP1_R435<br><br>HIST2H3A;H3F3B;H3F3A;HIST3H3;HIST1H3A;HIST2H3PS2;H3F3C_R80<br><br>HNRNPA1_R206<br><br>HNRNPAB_R270<br><br>HNRNPU_R733, R739<br><br>HNRNPUL1_R529, R531<br><br>IFT57_R259<br><br>NUTM1_R205, R208, R215<br><br>PABPC1_R448<br><br>PAGE5_R15<br><br>SAFB_R648<br><br>SAFB2_R903<br><br>STARD9_R929<br>272;274;277;929;931<br><br>TAF15_R567, R203, R182, R184, R525, R532	HNRNPA1<br>HNRNPA2B1<br>SQSTM1

CD55<br>CD59<br>CDKN2A<br>CELF2;CELF1<br>CEP131<br>CEP152<br>CFL1<br>CLINT1<br>COPA<br>CORO1C<br>CPNE8<br>CSNK1A1<br>DAB2<br>DARS<br>DBN1<br>DDX1<br>DDX17<br>DDX21<br>DDX39A;DDX39;DDX39B;hCG<br>DDX3X;DDX3Y<br>DDX47<br>DDX5<br>DDX54<br>DHX30<br>DHX9<br>DLST<br>DNAJA1<br>DSG2<br>DSP<br>EEF1A1P5;EEF1A1;EEF1A2<br>EEF1B2<br>EEF1D<br>EEF1G<br>EEF2<br>EIF2S3L;EIF2S3<br>EIF4A1;EIF4A2<br>EIF4A3;EIF4A1;EIF4A2<br>ELP6<br>EMD<br>FAM120A<br>FAM120B<br>FAM98A<br>FASN<br>FBL;FBLL1<br>FHL2<br>FLII<br>FLNA<br>FLNB<br>FLNC<br>FOLR1<br>FTH1<br>FUSIP1;SRSF10<br>FXR1<br>G3BP1<br>G3BP2<br>GAPDH<br>GEMIN4;RTTN<br>GLG1<br>GLIPR2<br>GNAI2	SLC25A5;SLC25A6;SLC25A4<br>SQSTM1<br>SRSF7<br>SSBP1<br>SSFA2<br>TPD52<br>TRIM16<br>TUBA1B;TUBA1C;TUBA1A;TUBA3C;KLK9<br>TUBB;TUBB4B;TUBB2B;TUBB2A;TUBB4A;TUBB3;TUBB8<br>TUBG2;TUBG1<br>UBB;RPS27A;UBC;UBA52;UBBP4			R251	182;184;189;203;525;532;559;567<br><br>TRA2B_R141	

GNAI3<br>GNB1<br>GPC1<br>GULP1<br>H1FX<br>HADHA<br>HADHB<br>HIP1<br>HIST1H1B<br>HIST1H1C<br>HIST1H1E;HIST1H1D<br>HIST1H2AC;HIST3H2A;HIST1H2AB<br>HIST1H2BN;HIST1H2BL;HIST1H2BM;HIST1H2BH;HIST2H2BF;HIST1H2BC;HIST1H2BD;H2BFS;HIST1H2BK<br>HIST1H4A<br>HIST2H3A;H3F3B;H3F3A;HIST3H3;HIST1H3A;HIST2H3PS2;H3F3C<br>HNRNPA1<br>HNRNPA2B1<br>HNRNPA3<br>HNRNPAB<br>HNRNPC<br>HNRNPF<br>HNRNPH1;HNRNPH2<br>HNRNPK<br>HNRNPL<br>HNRNPM<br>HNRNPR;SYNCRIP<br>HNRNPU<br>HNRNPUL1<br>HSP90AB1<br>HSPA1A<br>HSPA5<br>HSPA8<br>HSPA9<br>HSPB1<br>HSPD1<br>HTRA1<br>IARS<br>IFT57<br>IL17REL<br>ILF3<br>IMPDH2<br>IPO7<br>IPO9<br>ITPR1<br>ITPR3<br>JUP<br>KARS<br>KHDRBS1<br>KIF14<br>KIF23<br>KPNB1<br>KRT18<br>L3MBTL3						

LGALS8<br>LIMCH1<br>LMNA<br>LMO7<br>LONP2<br>LRRFIP2<br>LUZP1<br>MAP4K4<br>MAP7<br>MATR3<br>MCM7<br>MISP<br>MPRIP<br>MYH10<br>MYH9<br>MYL9<br>MYO18A<br>MYO1B<br>MYO6<br>MZT2B<br>NAP1L4;NAP1L1<br>NAT10<br>NCL<br>NONO<br>NPM1<br>NUMB<br>NUTM1<br>OGDH;OGDHL<br>OGT<br>PABPC1<br>PABPC4<br>PACSIN3<br>PAGE5<br>PALLD<br>PARVA<br>PCM1<br>PFKP<br>PHGDH<br>PIBF1<br>PIK3C2A<br>PKM<br>PLEC<br>POU2F2;KPNA2<br>PPL<br>PPP1CB<br>PPP1R12A<br>PPP2R2A<br>PRKAR1A<br>PRKCI<br>PRKDC<br>PRPF8<br>PSMC4<br>PTRF<br>RACGAP1<br>RAI14<br>RBBP4<br>RBM14<br>RBMX;RBMXL1<br>RCC2<br>RPL10;RPL10L<br>RPL10A<br>RPL11<br>RPL12<br>RPL13						

RPL15<br>RPL17<br>RPL18<br>RPL18A<br>RPL19<br>RPL21<br>RPL22<br>RPL26;KRBA2;RPL26L1<br>RPL27<br>RPL27A<br>RPL28<br>RPL3<br>RPL30<br>RPL31<br>RPL34<br>RPL35<br>RPL36<br>RPL39P5;RPL39<br>RPL4<br>RPL6<br>RPL7<br>RPL7A<br>RPL8<br>RPS11<br>RPS13<br>RPS14<br>RPS16<br>RPS17;RPS17L<br>RPS18<br>RPS19<br>RPS2<br>RPS20<br>RPS23<br>RPS25<br>RPS27<br>RPS3<br>RPS3A<br>RPS4X;RPS4Y2;RPS4Y1<br>RPS6<br>RPS8<br>RPS9<br>RTCB<br>RUVBL1<br>RUVBL2<br>SAFB<br>SAFB2<br>SERBP1<br>SFN<br>SLC25A11<br>SLC25A3<br>SLC25A5;SLC25A6;SLC25A4<br>SLTM<br>SPATS2L<br>SPECC1<br>SPECC1L;SPECC1L-ADORA2A<br>SPTAN1<br>SPTBN1<br>SPTBN2<br>SQSTM1<br>SRP14<br>SRSF6;SRSF5						

;SRSF4<br>SRSF7<br>SSBP1<br>SSFA2<br>STARD9<br>SVIL<br>TAF15<br>TCP1<br>TES<br>TGM2<br>THY1<br>TMEM160<br>TPD52<br>TPM4<br>TRA2B<br>TRIM16<br>TRIM21<br>TRIM56<br>TRIP6<br>TRMT1;LIMA1<br>TUBA1B;TUBA1C;TUBA1A;TUBA3C;KLK9<br>TUBA4A;TUBA8<br>TUBB;TUBB4B;TUBB2B;TUBB2A;TUBB4A;TUBB3;TUBB8<br>TUBG2;TUBG1<br>TUBGCP2<br>TUBGCP3<br>TUBGCP6<br>UBB;RPS27A;UBC;UBA52;UBBP4<br>VAPA<br>VIM<br>WDR1<br>XIAP<br>XRCC5<br>YBX1;YBX3<br>YTHDC1<br>YWHAE<br>YWHAG<br>YWHAH<br>YWHAQ<br>YWHAZ						

Supplementary Table 1, related to Figure 1. Putative C9ORF72-associated proteins identified by LC-MS/MS in arsenite-treated HeLa cells. See also Supplementary Data 1.

Supplementary Table 2. Gene ontology terms based on analysis of C9ORF72 proteome upon oxidative stress

GO Category	GO Term	Corrected *p*-Value	Cluster Frequency
Molecular function	RNA binding<br>Nucleic acid binding<br>Nucleotide binding<br>rRNA binding<br>Single-stranded RNA binding<br>Ribonucleotide binding	4.7131E-9<br>8.0499E-6<br>2.5634E-5<br>6.5744E-4<br>1.7181E-2<br>2.4969E-2	36.3%<br>56.8%<br>45.4%<br>6.8%<br>4.5%<br>27.2%
Cellular Component	Cytosolic ribosome<br>Non-membrane bound organelle<br>Ribonucleoprotein complex<br>Macromolecular complex<br>Cytosolic small ribosomal subunit<br>Spliceosomal complex	4.2193E-11<br>4.3547E-11<br>4.3547E-11<br>2.3891E-10<br>4.9731E-7<br>1.8059E-4	20.9%<br>62.7%<br>34.8%<br>67.4%<br>11.6%<br>11.6%
Biological process	Translation elongation<br>Cellular macromolecular complex assembly<br>Protein polymerization<br>Cellular macromolecular complex organization<br>Translation<br>Cellular protein complex assembly<br>RNA splicing<br>mRNA processing<br>Protein complex assembly<br>Nuclear mRNA splicing via spliceosome	6.8141E-11<br>2.7869E-7<br>4.6117E-7<br>4.8840E-7<br>8.0371E-7<br>2.1154E-4<br>3.3182E-3<br>4.5402E-3<br>9.5164E-3<br>3.1006E-2	23.8%<br>26.1%<br>14.2%<br>26.1%<br>23.8%<br>14.2%<br>14.2%<br>14.2%<br>16.6%<br>7.1%

Supplementary Table 2, related to Figure 1. Gene ontology terms based on analysis of C9ORF72 interactome upon oxidative stress.

Supplementary Table 3. Comparison of p62/SQSTM1 interactome with and without arsenite treatment

p62 Interactors -Arsenite		p62 Interactors +Arsenite		Common p62 Interactors + and - Arsenite	
SAINTq Analysis BFDR < 0.05	Saint BFDR < 0.05 and enriched in all 3 p62 IPs	SAINTq Analysis BFDR < 0.05	Saint BFDR < 0.05 and enriched in all 3 p62 IPs	SAINTq Analysis BFDR < 0.05	Saint BFDR < 0.05 and enriched in all 3 p62 IPs
ACIN1	ACTB	ACTB	ACTB	ACTB	ACTB
ACTB	AKR1C1	AKR1C1	AKR1C1	AKR1C1	AKR1C1
AKR1C1	ALYREF	ANXA2;ANXA2P2	ANXA2;ANXA2P2	ANXA2;ANXA2P2	ANXA2;ANXA2P2
ALDOA	ANXA2;ANXA2P2	BCLAF1	BCLAF1	BCLAF1	BCLAF1
ALYREF	ATAD3A	CASC3	CASC3	COL5A1	COL5A1
ANXA2;ANXA2P2	ATXN2L	COL5A1	COL5A1	DDX1	DDX1
ATAD3A	BCLAF1	COLGALT1	COLGALT1	ERH	ERH
ATXN2L	COL5A1	DDX1	DDX1	FXR2	HSPA1A
BCLAF1	CRTC2	DSC1	EEF1D	HIST1H4A	HSPA5
COL5A1	DDX1	EEF1A1P5;EEF1A	ERH	HIST2H2AC;HIST2	HSPA8
CRTC2	DDX3X;DDX3Y	1;EEF1A2	GAPDH	H2AA3;HIST1H2AJ	HSPA9
CSE1L	DHX9	EEF1D	HIST1H2AC;HIST3	;HIST1H2AH;H2AF	LMNA
DDX1	EIF4A1;EIF4A2	ERH	H2A;HIST1H2AB;H	J;HIST1H2AD;HIS	MAP1B
DDX3X;DDX3Y	ELAVL1	FXR2	IST1H2AA	T1H2AG	MVP
DHX9	EMD	GAPDH	HIST1H2BK;HIST1	HSP90AB1	MYH9
EEF1G	ERH	HIST1H2AC;HIST3	H2BN;H2BFS;HIST	HSPA1A	PABPC1;PABPC3
EIF4A1;EIF4A2	FAM120A	H2A;HIST1H2AB;H	1H2BL;HIST1H2B	HSPA5	PDCD6
EIF4A3	FXR1	IST1H2AA	M;HIST1H2BH;HIS	HSPA8	RPA1
ELAVL1	G3BP1	HIST1H2BK;HIST1	T2H2BF;HIST1H2B	HSPA9	RPS18
EMD	HNRNPA2B1	H2BN;H2BFS;HIST	C;HIST1H2BD	LMNA	RPS27A;UBB;UBC
ERH	HNRNPK	1H2BL;HIST1H2B	HIST1H4A	MAP1B	;UBA52;UBBP4
FAM120A	HNRNPU	M;HIST1H2BH;HIS	HIST2H3A;HIST3H	MVP	SQSTM1
FTH1	HSPA1A	T2H2BF;HIST1H2B	3;H3F3B;H3F3A;HI	MYH9	SRSF3
FXR1	HSPA5	C;HIST1H2BD	ST2H3PS2;H3F3C	NUFIP2	SRSF7
FXR2	HSPA8	HIST1H4A	HSPA1A	PABPC1;PABPC3	THRAP3
G3BP1	HSPA9	HIST2H2AC;HIST2	HSPA5	PDCD6	TRA2B
HIST1H4A	HSPB1	H2AA3;HIST1H2AJ	HSPA8	PDE6H;MYL6	
HIST2H2AC;HIST2	IGF2BP1	;HIST1H2AH;H2AF	HSPA9	PPIA	
H2AA3;HIST1H2AJ;	IGF2BP3	J;HIST1H2AD;HIS	ILF2	RPA1	
HIST1H2AH;H2AFJ;	ILF3	T1H2AG	LMNA	RPS18	
HIST1H2AD;HIST1	IMMT	HIST2H3A;HIST3H	MAP1B	RPS27A;UBB;UBC	
H2AG	KHDRBS1	3;H3F3B;H3F3A;HI	MVP	;UBA52;UBBP4	
HNRNPA1;HNRNP	LARP1	ST2H3PS2;H3F3C	MYH9	SQSTM1	
A1L2	LMNA	HNRNPR	NDUFV1	SRSF3	
HNRNPA2B1	MAP1B	HRNR	NUFIP2	SRSF7	
HNRNPK	MOV10	HSP90AB1	PABPC1;PABPC3	THRAP3	
HNRNPU	MVP	HSPA1A	PDCD6	TRA2B	
HSP90AB1	MYH9	HSPA5	PPIA	TUBA1B	
HSPA1A	NCKAP5L	HSPA8	PRDX1		
HSPA5	NCL	HSPA9	RBMX;RBMXL1		
HSPA8	PABPC1;PABPC3	ILF2	RPA1		
HSPA9	PABPC4	LMNA	RPL8		
HSPB1	PDCD6	MAP1B	RPS11		
HSPD1	PDE6H;MYL6	MVP	RPS18		
IGF2BP1	PFN1	MYH9	RPS27A;UBB;UBC		
IGF2BP3	PKM	NDUFV1	;UBA52;UBBP4		
ILF3	PLOD1	NFATC1	SNRPD1		
IMMT	RPA1	NUFIP2	SQSTM1		
KHDRBS1	RPA3	PABPC1;PABPC3	SRSF10		
LARP1	RPL17	PDCD6	SRSF3		
LMNA	RPL23	PDE6H;MYL6	SRSF7		
MAP1B	RPL30	PLOD3	THRAP3		
MOV10	RPL39P5;RPL39	PPIA	TKT		
MVP	RPL7A	PRDX1	TRA2A		
MYH9	RPLP0;RPLP0P6	PURA	TRA2B		
NCKAP5L	RPLP2	RBMX;RBMXL1	TUBA1B		
NCL	RPS18	RPA1			
NUFIP2	RPS25	RPL8			

PABPC1;PABPC3<br>PABPC4<br>PDCD6<br>PDE6H;MYL6<br>PFN1<br>PKM<br>PLOD1<br>PPIA<br>RPA1<br>RPA3<br>RPL17<br>RPL23<br>RPL30<br>RPL39P5;RPL39<br>RPL7<br>RPL7A<br>RPLP0;RPLP0P6<br>RPLP2<br>RPS18<br>RPS25<br>RPS27A;UBB;UBC;UBA52;UBBP4<br>RPS3<br>RPS3A<br>RPS4X<br>RPS9<br>SQSTM1<br>SRP14<br>SRRM2<br>SRSF1<br>SRSF3<br>SRSF6<br>SRSF7<br>SSBP1<br>SYNCRIP<br>THRAP3<br>TRA2B<br>TUBA1B<br>TUBB<br>UPF1<br>VIM<br>XRCC6<br>ZC3HAV1	RPS27A;UBB;UBC;UBA52;UBBP4<br>RPS3<br>RPS3A<br>RPS9<br>SQSTM1<br>SRP14<br>SRRM2<br>SRSF1<br>SRSF3<br>SRSF6<br>SRSF7<br>SSBP1<br>SYNCRIP<br>THRAP3<br>TRA2B<br>TUBB<br>VIM<br>XRCC6	RPS11<br>RPS18<br>RPS27A;UBB;UBC;UBA52;UBBP4<br>SNRPD1<br>SQSTM1<br>SRSF10<br>SRSF3<br>SRSF7<br>THRAP3<br>TKT<br>TRA2A<br>TRA2B<br>TUBA1B			

Supplementary Table 3, related to Figure 1. Comparison of p62/SQSTM1 interactome with and without arsenite treatment. See also Supplementary Data 2.

Supplementary Table 4. Gene ontology terms based on analysis of putative p62/SQSTM1 interactors in arsenite-treated HeLa cells

GO Category	GO Term	Corrected *p*-Value	Cluster Frequency
Molecular function	RNA binding	1.5706E-6	29.4%
	Nucleic acid binding	5.2963E-5	52.9%
	Nucleotide binding	7.3112E-4	39.2%
	Unfolded protein binding	1.1727E-3	9.8%
	rRNA binding	2.7362E-3	5.8%
	Protein binding	3.0936E-3	78.4%
Cellular Component	Macromolecular complex	1.6345E-11	68.0%
	Ribonucleoprotein complex	4.5913E-11	32.0%
	Non-membrane bound organelle	5.8026E-7	48.0%
	Cytosolic ribosome	3.5825E-5	10.0%
	Spliceosomal complex	4.0265E-2	6.0%
Biological process	Cellular macromolecular complex assembly	5.6645E-9	26.0%
	Cellular macromolecular complex subunit organization	2.0836E-8	26.0%
	mRNA metabolic process	2.3421E-6	22.0%
	RNA splicing	2.3421E-6	20.0%
	mRNA processing	4.3838E-6	20.0%
	Translation elongation	4.0521E-5	12.0%
	Nuclear mRNA splicing via spliceosome	4.3680E-4	10.0%
	Macromolecule metabolic process	2.4346E-3	54.0%
	Spliceosome assembly	4.7159E-3	6.0%
	Translation	1.0676E-2	12.0%
	Response to unfolded protein	2.9928E-2	6.0%
	Ribonucleoprotein complex assembly	4.4298E-2	6.0%

Supplementary Table 4, related to Figure 1. Gene ontology terms significantly enriched in the putative p62/SQSTM1 interactome in cells treated with arsenite. Putative p62 interactors with SAINT FDR < 0.01 and enriched in all three immunoprecipitations were used.

Supplementary Table 5. Putative p62/SQSTM1 interactors in arsenite-treated HeLa cells

P62/SQSTM1 Putative Interactors upon Oxidative Stress			
Stress Granule Components[1]	Di-methylated Arginine Proteins	Potential PRMT5 Substrates[4]	Di-methylated R sites interacting with p62
CASC3<br>DDX1<br>HSPA9<br>LMNA<br>NUFIP2<br>PABPC1<br>PRDX1	BCLAF1<br>HIST3H3<br>HSPA1A<br>HSPA8<br>ILF2<br>PABPC1;PABPC3<br>RBMX;RBMXL1<br>THRAP3<br>TRA2B	ALYREF_R45<br><br>FUS_R218, R259, R394<br><br>HNRNPA0_R139, R291<br><br>HNRNPA1_R206, R218, R232, R225, R194, R213, R196, R215, R265<br><br>HNRNPA1L2_R194, R196<br><br>HNRNPU_R715, R720, R709, R755, R572<br><br>HNRNPUL1_R181, R620<br><br>KHDRBS1_R304, R320<br><br>NDUFV1_R88<br><br>RBMX;RBMXL1_R144<br><br>SFPQ_R25<br><br>TAF15_R373, R293, R206, R286, R185, R338, R308, R315, R331, R195, R301, R124, R348, R137, R234<br><br>THRAP3_R101, R825<br><br>TRA2B_R224, R238, R251	ALYREF_R45, R70, R204<br><br>FUS_R216, R218<br><br>HIST2H3A;HIST3H3;H3F3B;H3F3A;HIST2H3PS2;H3F3C_R37<br><br>HNRNPA0_R291<br><br>HNRNPA1;HNRNPA1L2_R206; R194, R196<br><br>HNRNPU_R733, R739<br><br>HNRNPUL1_R529, R531<br><br>KHDRBS1_R325<br><br>MYH9_R1145<br><br>NDUFV1_R28, R36<br><br>PABPC1;PABPC3_R493<br><br>PABPC4_R489<br><br>SFPQ_R19, R25<br><br>TAF15_R525, R532<br><br>TRA2B_R241

Supplementary Table 5, related to Figure 1. p62/SQSTM1-associated proteins identified by LC-MS/MS in arsenite-treated HeLa cells. Putative p62 interactors with SAINT FDR < 0.01 and enriched in all three immunoprecipitations were used.

Supplementary Table 6. Common components of the putative C9ORF72 and p62/SQSTM1 interactomes in arsenite-treated HeLa cells and gene ontology analyses of these common components

Putative interactors of both C9ORF72 and p62/SQSTM1 in arsenite-treated HeLa cells					
Putative interactors of both C9ORF72 and p62	Stress Granule Components[1]	Di-methylated Arginine Proteins	Potential PRMT5 Substrates[4]	Di-methylated R sites	ALS-linked Proteins[2]
HIST1H2AC;HIST3H2A;HIST1H2AB<br>HIST1H2BN;HIST1H2BL;HIST1H2BM;HIST1H2BH;HIST2H2BF;HIST1H2BC;HIST1H2BD;H2BFS;HIST1H2BK<br>HIST1H4A<br>HSPA8<br>MYH9<br>PABPC1<br>RBMX;RBMXL1<br>RPS11<br>RPS18<br>RPS27A;UBB;UBC;UBA52;UBBP4<br>SQSTM1<br>SRSF7	PABPC1	HSPA8<br>PABPC1<br>RBMX;RBMXL1	FUS_R218, R259, R394<br><br>HNRNPA1_R206, R218, R232, R225, R194, R213, R196, R215, R265<br><br>HNRNPU_R715, R720, R709, R755, R572<br><br>HNRNPUL1_R181, R620<br><br>RBMX;RBMXL1_R144<br><br>TAF15_R373, R293, R206, R286, R185, R338, R308, R315, R331, R195, R301, R124, R348, R137, R234<br><br>TRA2B_R224, R238, R251	FUS_R216, R218<br><br>HNRNPA1_R206<br><br>HNRNPU_R733, R739<br><br>HNRNPUL1_R529, R531<br><br>TAF15_R525, R532	SQSTM1

Gene ontology analyses of Putative interactors of both C9ORF72 and p62/SQSTM1 in arsenite-treated HeLa cells			
GO Catergory	GO Term	Corrected *p*-Value	Cluster Frequency
Molecular function	Structural constituent of ribosome<br>Nucleic acid binding<br>rRNA binding<br>RNA binding	1.0638E-3<br>1.6349E-3<br>7.0741E-3<br>1.0151E-2	25.0%<br>68.7%<br>12.5%<br>31.2%
Cellular Component	Macromolecular complex<br>Cytosolic small ribosomal subunit<br>Ribonucleoprotein complex<br>Cytosolic ribosome<br>Non-membrane bound organelle<br>Spliceosomal complex	2.1212E-7<br>5.6874E-7<br>3.0019E-6<br>7.1685E-6<br>1.1804E-5<br>2.0259E-2	87.5%<br>25.0%<br>43.7%<br>25.0%<br>68.7%<br>12.5%
Biological process	Cellular macromolecular complex subunit organization<br>Translation elongation<br>Translation<br>RNA splicing<br>mRNA processing	4.5774E-5<br>8.8696E-5<br>4.3873E-3<br>3.3178E-2<br>3.7218E-2	37.5%<br>25.0%<br>25.0%<br>18.7%<br>18.7%

Supplementary Table 6, related to Figure 1. Gene ontology analyses of shared components of the putative C9ORF72 and p62/SQSTM1 interactomes in arsenite-treated HeLa cells. Putative interactors with SAINT FDR < 0.01 and enriched in all three immunoprecipitations were used.

Supplementary Table 7. Symmetric arginine dimethylation sites detected on FUS

Amino acid position	Method of detection (MaxQuant, FDR < 0.01)
R216, R218 and R503	Mass spectrometric analysis of immuno-precipitated FUS with and without PRMT5 depletion; summarized dimethyl (R) sites were enriched more in FUS immuno-precipitates in the presence of PRMT5
R218, R371, R394 and R407	Mass spectrometric analysis of FUS following its *In vitro* methylation assay with and without purified PRMT5/MEP50; summarized dimethyl (R) sites were enriched in the presence of PRMT5/MEP50
R218	Western blotting immuno-enriched R218K point mutant form of FUS with an antibody that recognizes symmetrically dimethylated proteins

Supplementary Table 7, related to Figure 5. Symmetric arginine dimethylation sites on FUS identified by MaxQuant analysis of LC-MS/MS. See also Supplementary Data 3.

Supplementary Table 8. Information of *p62 WT* and *p62*$^{-/-}$ mice used in the study

Genotype	Case Number	Sex	Age	Application
p62 WT	1	M	35-36w	Splicing (Fig. 5i)
	2	F	24w +0d	Splicing (Fig. 5i)
	3	F	33w +0d	Splicing (Fig. 5i); Co-IPs (Supp. Fig. 5i)
	4	F	7w +0d	Cerebellum - WB (Fig. 5g)
	5	F	3w +0d	Cerebellum - WB (Fig. 5g)
	6	F	9w +0d	Cerebellum - WB (Fig. 5g)
	7	F	31w +0d	Hippocampus - WB (Fig. 5g)
	8	F	31w +0d	Hippocampus - WB (Fig. 5g)
p62 KO	1	M	37w +5d	Splicing (Fig. 5i)
	2	F	37w +5d	Splicing (Fig. 5i)
	3	M	42w +3d	Splicing (Fig. 5i); Co-IPs (Supp. Fig. 5i)
	4	F	7w +0d	Cerebellum - WB (Fig. 5g)
	5	F	3w +0d	Cerebellum - WB (Fig. 5g)
	6	F	9w +0d	Cerebellum - WB (Fig. 5g)
	7	M	26w +3d	Hippocampus - WB (Fig. 5g)
	8	F	6w +6d	Hippocampus - WB (Fig. 5g)

Supplementary Table 9. Antisense sequences of siRNAs used in the study

Target	Thermo Fisher Scientific: Silencer® Select ID and Catalog #
Negative Control (Ctl)	4390847
ATG5 #1	s18160 - 4392420; 5' – UAUCUCAUCCUGAUAUAGCgt – 3'
ATG5 #2	s18158 - 4392420; 5' – UAGAGCUUCAAUUGCAUCCtt – 3'
ATG7 #1	s20650 - 4392420; 5' – UGGUGUUAUACAGUGUUCCaa – 3'
ATG7 #2	s20651 - 4392420; 5' – UGAACUCCAAUGUUAAGCGag – 3'
C9ORF72 #1	s47490 - 4392420; 5' – UAUGCAUCCAUAUUCUUCCtt – 3'
C9ORF72 #2	s47491 - 4392420; 5' – UUUCCAUCAAAGAUUAAUGaa – 3'
FUS	s5403 - 4392420; 5' – AAUUGUAACAUUCUCACCCag – 3'

Fus	s107707 - 4390771; 5' – UUUACCAUCAAACCAGUCGat – 3'
FUS-3'UTR	5' – UUGGGUGAUCAGGAAUUGGaa – 3'
NBR1	s57876 - 4392420; 5' – UAUGAUACUGCACCAGACCcg – 3'
OPTN	s19720 - 4392420; 5' – UUGAGUGCAACUUCAAGUCtc – 3'
P62 #1	s16960 - 4392420; 5' – UCUUUUCCCUCCGUGCUCCac – 3'
P62 #2	s16962 - 4392420; 5' – UUUAAUGUAGAUUCGGAAGat – 3'
PRMT5	s20375 - 4390824; 5' – AGAGUUCAUAGGCAUUAGGtg – 3'
SMN	s231507 - 4390827; 5' – UCCAAUAUCAUUCAAAAUCta – 3'

Supplementary Table 10. Plasmids used in the study

**Name**	**Manufacturer**
FLAG/HA-FUS	Addgene (26374)
FLAG/HA-FUS-R521G	Addgene (26380)
FLAG/HA-FUS-R521H	Addgene (26381)
FLAG/HA-FUS-R216C	Created by PCR from FLAG/HA-FUS
FLAG/HA-FUS-R218K	Created by PCR from FLAG/HA-FUS
FLAG/HA-FUS-R216C+R218K	Created by PCR from FLAG/HA-FUS
GFP-DDX3	Subcloned DDX3 into pcDNA 3.1 GFP plasmid
GFP-P62	[8]
GFP-P62ΔUBA	[8]
GFP-P62ΔLIR	[8]
GFP-SMN	Subcloned from pcDNA3.1-Myc-SMN [9]
GFP-SMNΔTdr	Subcloned from GFP-SMN
pGST2	[10]
GST-FUS	Addgene (44978)
HA-P62	Addgene (28027)
Myc-DDK-C9ORF72	OriGene (RC209700)
Myc-SMN	[9]
pLKO.1-sh(Luciferase)	TRC (The RNAi Consortium)

pLKO.1-sh(PRMT5)	TRC (The RNAi Consortium)

Supplementary Table 11. Sequences of primer pairs used in the study

**Target gene / locus**		**Sequence (5' – 3')**	**Application**
18S rRNA	Fwd	GTAACCCGTTGAACCCCATT	Transcript-specific qRT-PCR primers
	Rev	CCATCCAATCGGTAGTAGCG	
C9ORF72 All	Fwd	CCCACTTCATAGAGTGTGTGTTG	
	Rev	TTCCATTCTCTCTGTGCCTTC	
C9ORF72 Short	Fwd	GAAATCACACAGTGTTCCTGAAGAA	
	Rev	ATCTGCTTCATCCAGCTTTTATGA	
C9ORF72 Long	Fwd	CATGGCTCAGGATACGATCA	
	Rev	GGAAGGCTTTCACTAGAGTGTCTC	
GAPDH	Fwd	ATCTTCTTGTGCAGTGCCAG	
	Rev	TTTGCCACTGCAAATGGCAG	
p62/ Sqstm1	Fwd	AGATGCCAGAATCGGAAGGG	
	Rev	GAGAGGGACTCAATCAGCCG	
PRMT5	Fwd	GGAACTCTGAAGCGGCTATG	
	Rev	TGATTAGACGGGAGGTCAGC	
Zcchc6/ Tut7	Fwd	GCCCACAGTTCAAAGGCTCT	Splice variant-specific RT-PCR primers
	Rev	GTGTCCTGTGCCATGAACCT	
FUS-R216C	Fwd	ACCGTGGAGGCTGCGGCAGGGGT	FUS mutant-specific PCR primers
	Rev	ACCCCTGCCGCAGCCTCCACGGT	
FUS-R218K	Fwd	GGAGGCCGCGGCAAGGGTGGCAGT	
	Rev	ACTGCCACCCTTGCCGCGGCCTCC	
FUS-216C+R218K	Fwd	CCGTGGAGGCTGCGGCAAGGGTGGCAGT	
	Rev	ACTGCCACCCTTGCCGCAGCCTCCACGG	

Supplementary Table 12. Antibodies used in the study

Target	Manufacturer	Application
Dimethyl-Arginine, asymmetric – ASYM24/25	[11,12]	WB, IF
C9ORF72	Santa Cruz Biotechnology (sc-138763)	WB, IF, PLA, IP/MS
C9ORF72	Proteintech (22637-1-AP)	WB, IF, Co-IP
C9ORF72	Proteintech (66140-1-Ig)	IF
C9ORF72 Long	[13]	WB
DDX3	Bethyl Laboratories (A300-474A)	WB, IF
EWS	Bethyl Laboratories (A300-417A)	WB
FMRP	EMD Millipore (MAB2160)	IF, EM
FUS	Bethyl Laboratories (A300-302A)	WB, Co-IP, IF, PLA, EM, IP/MS
GAPDH	BioLegend (919501)	WB
GST	[14]	WB
HA	EMD millipore (05-904)	WB, Co-IP, IF, PLA, IP/MS
HuR	Santa Cruz Biotechnology (sc-5261)	IF
LC3	Sigma-Aldrich (L7543)	WB, IF, PLA
LC3	Cell Signaling Technology (3868S)	WB, IF, PLA
Myc	ATCC Hybridoma (9E10)	WB
NBR1	Abnova (H00004077-M01)	IF, PLA, WB
Optineurin	Abcam (ab23666)	IF, WB
P62	BD Transduction Laboratories (610833)	WB, IF, PLA, IHC, EM
P62	Sigma-Aldrich (P0067)	WB, Co-IP, IF, PLA
PABP	Santa Cruz Biotechnology (sc-32318)	IF
PRMT5	[15]	WB
Smith Antigen [Y12]	Hybridoma from Robin Reed (Harvard University, Boston, MA)	WB
SMN	BD Biosciences (610647)	WB, Co-IP
SMN	Santa Cruz Biotechnology (sc-7804)	IF
Dimethyl-Arginine, symmetric – SYM10/11	[12,15]	WB, Co-IP, IF

Dimethyl-Arginine, symmetric	Cell Signaling Technology (13222S)	IF
TDP-43	Sigma-Aldrich (T1580)	IF
TIAR	Cell Signaling Technology (8509S)	IF
Tubulin	Thermo Fisher Scientific (PA5-22060)	WB
Ubiquitin	Cell Signaling Technology (3936S)	IF, WB
IgG mouse	eBioscience (14-4714-82)	Co-IP, IF, PLA, IP/MS
IgG rabbit	GeneTex (GTX35035)	Co-IP, IF, PLA, IP/MS
HRP secondary	Jackson ImmunoResearch (111-035-144, 115-035-174); GenScript (A00178)	WB
Fluorescent secondary	Invitrogen (Alexa Fluor® Dyes)	IF

Supplementary References

1 Jain, S. *et al.* ATPase-Modulated Stress Granules Contain a Diverse Proteome and Substructure. *Cell* **164**, 487-498, doi:10.1016/j.cell.2015.12.038 (2016).

2 Abel, O., Powell, J. F., Andersen, P. M. & Al-Chalabi, A. ALSoD: A user-friendly online bioinformatics tool for amyotrophic lateral sclerosis genetics. *Human mutation* **33**, 1345-1351, doi:10.1002/humu.22157 (2012).

3 Bremang, M. *et al.* Mass spectrometry-based identification and characterisation of lysine and arginine methylation in the human proteome. *Molecular bioSystems* **9**, 2231-2247, doi:10.1039/c3mb00009e (2013).

4 Larsen, S. C. *et al.* Proteome-wide analysis of arginine monomethylation reveals widespread occurrence in human cells. *Science signaling* **9**, rs9, doi:10.1126/scisignal.aaf7329 (2016).

5 Uhlmann, T. *et al.* A method for large-scale identification of protein arginine methylation. *Molecular & cellular proteomics : MCP* **11**, 1489-1499, doi:10.1074/mcp.M112.020743 (2012).

6 Sylvestersen, K. B., Horn, H., Jungmichel, S., Jensen, L. J. & Nielsen, M. L. Proteomic analysis of arginine methylation sites in human cells reveals dynamic regulation during transcriptional arrest. *Molecular & cellular proteomics : MCP* **13**, 2072-2088, doi:10.1074/mcp.O113.032748 (2014).

7 Guo, A. *et al.* Immunoaffinity enrichment and mass spectrometry analysis of protein methylation. *Molecular & cellular proteomics : MCP* **13**, 372-387, doi:10.1074/mcp.O113.027870 (2014).

8 Pankiv, S. *et al.* p62/SQSTM1 binds directly to Atg8/LC3 to facilitate degradation of ubiquitinated protein aggregates by autophagy. *The Journal of biological chemistry* **282**, 24131-24145, doi:10.1074/jbc.M702824200 (2007).

9 Bachand, F., Boisvert, F. M., Cote, J., Richard, S. & Autexier, C. The product of the survival of motor neuron (SMN) gene is a human telomerase-associated protein. *Molecular biology of the cell* **13**, 3192-3202, doi:10.1091/mbc.E02-04-0216 (2002).

10 Sheffield, P., Garrard, S. & Derewenda, Z. Overcoming expression and purification problems of RhoGDI using a family of "parallel" expression vectors. *Protein expression and purification* **15**, 34-39, doi:10.1006/prep.1998.1003 (1999).

11 Cote, J., Boisvert, F. M., Boulanger, M. C., Bedford, M. T. & Richard, S. Sam68 RNA binding protein is an in vivo substrate for protein arginine N-methyltransferase 1. *Molecular biology of the cell* **14**, 274-287, doi:10.1091/mbc.E02-08-0484 (2003).

12 Boisvert, F. M., Cote, J., Boulanger, M. C. & Richard, S. A proteomic analysis of arginine-methylated protein complexes. *Molecular & cellular proteomics : MCP* **2**, 1319-1330, doi:10.1074/mcp.M300088-MCP200 (2003).

13 Xiao, S. *et al.* Isoform-specific antibodies reveal distinct subcellular localizations of C9orf72 in amyotrophic lateral sclerosis. *Annals of neurology* **78**, 568-583, doi:10.1002/ana.24469 (2015).

14 Cote, J. & Richard, S. Tudor domains bind symmetrical dimethylated arginines. *The Journal of biological chemistry* **280**, 28476-28483, doi:10.1074/jbc.M414328200 (2005).

15 Boisvert, F. M. *et al.* Symmetrical dimethylarginine methylation is required for the localization of SMN in Cajal bodies and pre-mRNA splicing. *The Journal of cell biology* **159**, 957-969, doi:10.1083/jcb.200207028 (2002).

16 Vizcaino, J. A. *et al.* 2016 update of the PRIDE database and its related tools. *Nucleic acids research* **44**, D447-456, doi:10.1093/nar/gkv1145 (2016).
