# Supplementary material for: A complex of C9ORF72 and p62 uses arginine methylation to eliminate stress granules by autophagy
Source: Nat Commun. 2018 Jul 18;9:2794. doi: 10.1038/s41467-018-05273-7 (PMC6052026; doi:10.1038/s41467-018-05273-7)
Supplement: Supplementary file 2 — Description of Additional Supplementary Files [file 41467_2018_5273_MOESM2_ESM.pdf]

Description of Additional Supplementary Files

File Name: Supplementary Data 1
Description: Unprocessed output files generated by MaxQuant and SAINT analysis of LC-MS/MS results, related to Supplementary Table 1-2. C9ORF72 LC-MS/MS. File contains 3 Excel sheets: (i) protein groups interacting with C9ORF72 after arsenite treatment, (ii) dimethyl (KR) sites interacting with C9ORF72 after arsenite treatment and (iii) SAINT analysis of C9ORF72 interactors after arsenite treatment. Project accession: PXD009759

File Name: Supplementary Data 2
Description: Unprocessed output files generated by MaxQuant and SAINT analysis of LC-MS/MS results, related to Supplementary Table 3-5. p62 LC-MS/MS. File contains 4 Excel sheets: (i) protein groups interacting with HA-p62/SQSTM1 from cells with and without arsenite treatment, (ii) dimethyl (KR) sites interacting HA-p62/SQSTM1 from cells with and without arsenite treatment, (iii-iv) SAINT analysis of HA-p62/SQSTM1 interactors (iii) without and (iv) with arsenite treatment. Project accession: PXD009741

File Name: Supplementary Data 3
Description: Unprocessed output files generated by MaxQuant and SAINT analysis of LC-MS/MS results, related to Supplementary Table 7 - FUS Dimethyl(KR)Sites LC-MS/MS. File contains 3 Excel sheets: (i) dimethyl (KR) sites detected on FUS with and without PRMT5, with and without arsenite treatment of cells, (ii) dimethyl (KR) sites detected on FUS following its *in vitro* methylation by purified PRMT5/MEP50 complex and (iii) dimethyl (KR) sites detected on FUS following its *in vitro* methylation in the presence or absence of purified PRMT5/MEP50 complex. Project accession: PXD009760
